# Global Proposal for the TNM Staging System for Poorly Differentiated Thyroid Cancer

**DOI:** 10.1001/jamaoncol.2026.4705

**Published:** 2026-11-06

**Authors:** Xiao Shi, Qiaomu Liu, Daniel Scholfield, Dapeng Li, Livia Lamartina, Shuwei Chen, Hao Zhang, Alexander J. Papachristos, Omar Hamdy, Natalia Timofeeva, Amy Sheen, Xiangqian Zheng, Ming Song, Zhihong Wang, Xianying Meng, Lei Xie, Detao Yin, Ying Zheng, Shangtong Lei, Christoph Becker, Ekin Y. Koroglu, Fangzhou Liu, Jie Ming, Tianrun Liu, Hui Liu, Hengyu Li, Zhendong Li, Xingrui Li, Xinying Li, Jian Xu, Yijun Wu, Bo Zhang, Meiping Shen, Yansong Lin, Linzi Jia, Zhilong Ai, Dapeng Lei, Zhiyan Liu, Haixia Guan, Chuang Chen, Haipeng Xiao, Yihao Liu, Zhihui Li, Dongmei Ji, Qinghai Ji, Luis G. Carvajal-Carmona, Ana Patricia Estrada-Florez, Mabel E. Bohórquez, Matrix Man Him Fung, Martin Schlumberger, Lori Wirth, Louise Davies, Fabian Pitoia, Nelson Wohllk, João Martins Gama, Tommaso Porcelli, Domenico Salvatore, Mira Fink, Berna Evranos Ogmen, Eun Kyung Lee, Andrea Repaci, Giovanni Tallini, Cosimo Durante, Simone De Leo, Laura Fugazzola, Ilya Sleptsov, Young Joo Park, Mohamed-Amine Bani, Ian Ganly, Anthony J. Gill, Yu Wang

**Affiliations:** 1 Fudan University Shanghai Cancer Center, Department of Oncology, Shanghai Medical College, Fudan University, Shanghai, China; 2 Memorial Sloan Kettering Cancer Center, New York, New York; 3 Tianjin Medical University Cancer Institute and Hospital, Tianjin, China; 4 Gustave Roussy, Université Paris Saclay, ENDOCAN-TUTHYREF Network, Villejuif, France; 5 Sun Yat-sen University Cancer Center, Sun Yat-sen University, Guangzhou, China; 6 The First Hospital of China Medical University, Shenyang, China; 7 Royal North Shore Hospital, University of Sydney, Sydney, New South Wales, Australia; 8 Oncology Center, Mansoura University, Mansoura, Egypt; 9 N.I. Pirogov Clinic of High Medical Technologies, Saint Petersburg State University, Saint Petersburg, Russia; 10 The First Hospital of Jilin University, Changchun, China; 11 Sir Run Run Shaw Hospital, Zhejiang University School of Medicine, Hangzhou, China; 12 The First Affiliated Hospital of Zhengzhou University, Zhengzhou, China; 13 Jilin Cancer Hospital, Changchun, China; 14 Nanfang Hospital, Southern Medical University, Guangzhou, China; 15 Medical Center-University of Freiburg, Faculty of Medicine, University of Freiburg, Freiburg, Germany; 16 Ankara Bilkent City Hospital, Ankara Yildirim Beyazit University Faculty of Medicine, Ankara, Turkey; 17 Jiangsu Cancer Hospital, Nanjing, China; 18 Union Hospital, Tongji Medical College, Huazhong University of Science and Technology, Wuhan, China; 19 Sun Yat-sen Memorial Hospital, Sun Yat-sen University, Guangzhou, China; 20 Fujian Cancer Hospital, Fuzhou, China; 21 Shanghai Changhai Hospital, Naval Medical University, Shanghai, China; 22 Cancer Hospital of China Medical University, Shenyang, China; 23 Tongji Hospital, Tongji Medical College, Huazhong University of Science and Technology, Wuhan, China; 24 Xiangya Hospital, Central South University, Changsha, China; 25 Affiliated Tumor Hospital of Guangxi Medical University, Nanning, China; 26 The First Affiliated Hospital, Zhejiang University School of Medicine, Hangzhou, China; 27 China-Japan Friendship Hospital, Beijing, China; 28 The First Affiliated Hospital with Nanjing Medical University, Nanjing, China; 29 Peking Union Medical College Hospital, Beijing, China; 30 Shanxi Province Cancer Hospital, Taiyuan, China; 31 Zhongshan Hospital, Fudan University, Shanghai, China; 32 Qilu Hospital of Shandong University, Jinan, China; 33 Shanghai Sixth People’s Hospital, Shanghai Jiao Tong University School of Medicine, Shanghai, China; 34 Guangdong Provincial People’s Hospital, Southern Medical University, Guangzhou, China; 35 Renmin Hospital of Wuhan University, Wuhan, China; 36 The First Affiliated Hospital, Sun Yat-sen University, Guangzhou, China; 37 West China Hospital, Sichuan University, Chengdu, China; 38 The Health Equity Leadership, Science, and Community Research Laboratory, Genome Center, and Department of Biochemistry and Molecular Medicine, School of Medicine, University of California, Davis; 39 Grupo de Citogenética, Filogenia y Evolución de Poblaciones, Facultades de Ciencias y Facultad de Ciencias de la Salud, Universidad del Tolima, Ibagué, Colombia; 40 School of Clinical Medicine, Li Ka Shing Faculty of Medicine, The University of Hong Kong, Hong Kong SAR, China; 41 Massachusetts General Hospital, Mass General Brigham Cancer Institute, Harvard Medical School, Boston; 42 University of Wisconsin-Madison School of Medicine and Public Health, Madison; 43 Hospital de Clínicas, University of Buenos Aires, Buenos Aires, Argentina; 44 Hospital del Salvador, University of Chile, Santiago de Chile, Chile; 45 Hospitais Universitários de Coimbra, Unidade Local de Saúde de Coimbra, Coimbra, Portugal; 46 University of Naples Federico II, Naples, Italy; 47 Center for Thyroid Cancer, National Cancer Center, Goyang, Korea; 48 University of Bologna, IRCCS Azienda Ospedaliero-Universitaria di Bologna, Bologna, Italy; 49 Sapienza University of Rome, Rome, Italy; 50 ASST Santi Paolo e Carlo, Department of Health Sciences, University of Milan, Milan, Italy; 51 Seoul National University College of Medicine, Seoul, Korea

## Abstract

**Importance:**

Poorly differentiated thyroid cancer (PDTC) lacks a dedicated staging system, requiring use of the eighth version of the American Joint Committee on Cancer and Union for International Cancer Control TNM staging system (TNM-8) for differentiated thyroid cancer despite its distinct biology.

**Objective:**

To develop a PDTC-specific staging system.

**Design, Setting, and Participants:**

This cohort study analyzed data from the International Aggressive Thyroid Cancer Collaborative Group (IATCCG), including surgically treated patients at 91 centers in 14 countries from January 1984 to January 2026. External validation was performed using patients from the US Surveillance, Epidemiology, and End Results (SEER) database. Data were analyzed from December 2025 to June 2026.

**Main Outcomes and Measures:**

The primary end point was overall survival. With M1 disease predefined as stage IVB, the schema for stages I–IVA in patients with M0 disease were redefined. All possible TN combinations were fitted into multivariable Cox models, with the lowest Akaike information criterion defining the proposed system. Prognostic discrimination was assessed using adjusted hazard ratios (aHRs) and C statistics.

**Results:**

Among 1010 patients in the IATCCG cohort, 610 (60.4%) were female, and the median (IQR) age at diagnosis was 58 (46-68) years; median (IQR) follow-up was 41 (15-85) months. Among 170 patients in the SEER cohort, 85 (50.0%) were female, and the median (IQR) age at diagnosis was 61 (49-72) years; median (IQR) follow-up was 92 (42-154) months. In the IATCCG cohort, level VII lymph node metastasis (LNM) was associated with lower 5-year overall survival (45.2%; 95% CI, 33.4-61.2) than level VI (73.2%; 95% CI, 64.4-83.3) or lateral neck LNM (56.7%; 95% CI, 49.4-65.0). Level VII LNM was therefore reclassified from N1a to N1c. The lowest Akaike information criterion configuration among 16 738 candidates defined stages I to IVA as follows: I, T1N0/NX/N1a or T2N0/NX; II, T1N1b/N1c, T2N1a/N1b, or T3N0/NX/N1a; III, T2N1c, T3N1b, or T4aN0/NX/N1a; IVA, T3N1c, T4aN1b/N1c, or T4b any N; and IVB, M1 disease. Compared with TNM-8, stage I disease decreased from 44.4% (448 of 1010) to 24.6% (248 of 1010) and stage IV disease increased from 18.5% (187 of 1010) to 32.4% (327 of 1010) with the proposed system. The proposed staging had a higher C statistic than TNM-8 (0.74 [95% CI, 0.71-0.76] vs 0.71 [95% CI, 0.68-0.74]; difference, 0.03; 95% CI, 0.01-0.04). Corresponding SEER C statistic values were 0.76 (95% CI, 0.71-0.81) vs 0.74 (95% CI, 0.68-0.79), respectively (difference, 0.02; 95% CI, −0.01 to 0.06).

**Conclusions and Relevance:**

This cohort study found that this PDTC-specific staging system provides improved prognostic stratification reflecting its different biology. These data provide a strong rationale for the potential inclusion of a stand-alone American Joint Committee on Cancer staging system for PDTC.

## Key Points

**Question:**

Can a dedicated TNM staging system be developed for poorly differentiated thyroid cancer (PDTC)?

**Findings:**

In this cohort study of 1010 patients with PDTC, level VII lymph node metastasis was associated with significantly worse survival than level VI or lateral neck metastases, prompting its upgrading as a new N1c category and the establishment of a dedicated PDTC staging system with clear prognostic discrimination, which was externally validated by the Surveillance, Epidemiology, and End Results database.

**Meaning:**

A PDTC-specific TNM staging system is proposed to inform future staging revisions.

## Teaser

This cohort study evaluates the *American Joint Committee on Cancer Staging Manual*, *eighth edition *TNM staging protocol for poorly differentiated thyroid cancer and proposes a disease-specific staging system.

## Introduction

Poorly differentiated thyroid carcinoma (PDTC) is a rare and aggressive malignancy arising from thyroid follicular cells. Its definition has evolved over the years, and the tumor is currently defined by the World Health Organization according to the Turin consensus criteria.^1^ Although PDTC accounts for only 1% to 3% of all thyroid cancers, it constitutes the predominant cause of mortality among nonanaplastic thyroid malignant tumors.^3^ In terms of aggressiveness and lethality, PDTC lies between differentiated thyroid cancer (DTC) and anaplastic thyroid cancer, representing an independent biological entity.^1^

In thyroid oncology, the American Joint Committee on Cancer (AJCC) and Union for International Cancer Control (UICC) TNM staging system remains the criterion standard of risk stratification. However, owing to the paucity of robust data and inconsistent pathological criteria applied in the past, the eighth version of the AJCC/UICC TNM staging system (TNM-8) lacks a dedicated staging framework for PDTC. Clinically, while AJCC provides no specific staging guidance for PDTC,^10^ UICC advocates applying the DTC framework.^11^

Evidence shows the DTC staging framework inaccurately stratifies PDTC mortality risk,^8^ challenging the biological rationale for extrapolating specific DTC tenets to PDTC. Two issues appear particularly contentious: the 55-year age cutoff and classifying level VII (upper mediastinal) lymph node metastasis (LNM) as the mild N1a category. Given that level VII involvement is linked to poor outcomes in medullary thyroid cancer,^13^ labeling it as minor disease in aggressive PDTC seems clinically inadequate. The absence of a tailored, evidence-based PDTC staging system leaves clinicians without reliable tools for prognostic evaluation.

To address this critical gap, we established the International Aggressive Thyroid Cancer Collaborative Group (IATCCG) and assembled, to our knowledge, the largest global PDTC cohort to date, representing a multinational collaboration across 14 countries. Using this dataset, we sought to develop a novel PDTC-specific TNM staging system to provide a critical reference for the upcoming version nine of the AJCC TNM staging protocol.

## Methods

### Study Design and Participants

The study received ethical approval from the Institutional Review Board of the Fudan University Shanghai Cancer Center and from institutional review boards of participating centers, whenever required. Informed consent was waived due to the retrospective design, use of deidentified data per privacy laws, and absence of interventions or novel procedures, ensuring minimal risk to participants. This study is reported in compliance with the Strengthening the Reporting of Observational Studies in Epidemiology (STROBE) reporting guideline.

#### IATCCG Cohort

This international multicenter cohort included consecutive patients with PDTC who underwent initial surgery at 91 centers across 14 countries from January 1984 to January 2026 (China, US, Australia, Italy, France, Germany, Korea, Russia, Portugal, Turkey, Egypt, Chile, Argentina, and Colombia) (Table 1). This cohort was used for staging system derivation and internal evaluation. Detailed methodologies for pathological verification, patient exclusion, and TNM classification are provided in the eMethods in Supplement 1; the corresponding flowchart detailing selection process is presented in eFigure 1 in Supplement 1.

**Table 1.** Center-Level Distribution and Baseline Characteristics of the International Aggressive Thyroid Cancer Collaborative Group Cohort

Center	Total, No.	Sex, No. (%)		Median (IQR)	
		Female	Male	Age at diagnosis, y	Follow-up time, mo
Ankara Bilkent City Hospital, Ankara, Turkey	11	9 (81.8)	2 (18.2)	47 (40-60)	26.0 (19.5-42.5)
Gustave Roussy Cancer Campus, Villejuif, France	77	45 (58.4)	32 (41.6)	60 (51-72)	72.0 (27.0-119.0)
Hospital de Clínicas, University of Buenos Aires, Buenos Aires, Argentina	2	1 (50.0)	1 (50.0)	68 (61-74)	81.0 (64.5-97.5)
Hospital del Salvador, Universidad de Chile, Santiago, Chile	2	1 (50.0)	1 (50.0)	67 (64-70)	82.0 (60.5-103.5)
Maggiore Hospital, Bologna, Italy	12	3 (25.0)	9 (75.0)	64 (54-81)	50.5 (38.2-74.8)
Medical Center - University of Freiburg, Freiburg, Germany	11	6 (54.5)	5 (45.5)	64 (45-73)	43.1 (21.5-49.4)
Memorial Sloan Kettering Cancer Center, New York, New York	87	51 (58.6)	36 (41.4)	59 (44-70)	77.0 (40.5-139.5)
National Cancer Center, Goyang, Korea	15	11 (73.3)	4 (26.7)	57 (48-62)	30.0 (13.8-49.1)
Oncology Center, Mansoura University, Mansoura, Egypt	23	14 (60.9)	9 (39.1)	54 (42-64)	15.0 (5.5-56.0)
Royal North Shore Hospital, Sydney, New South Wales, Australia	92	46 (50.0)	46 (50.0)	65 (54-74)	55.5 (15.5-92.9)
Saint Petersburg State University Hospital, Saint Petersburg, Russia	45	30 (66.7)	15 (33.3)	56 (45-65)	74.0 (12.0-134.0)
Seoul National University Hospital, Seoul, Korea	57	39 (68.4)	18 (31.6)	60 (47-68)	69.2 (35.1-107.0)
IRCCS Azienda Ospedaliera Universitaria di Bologna-Policlinico di Sant’Orsola, Bologna, Italy	17	8 (47.1)	9 (52.9)	57 (53-69)	66.0 (25.3-147.6)
Unidade Local de Saúde de Coimbra, Coimbra, Portugal	6	4 (66.7)	2 (33.3)	53 (35-59)	96.0 (44.1-113.8)
University of Milan, Milan, Italy	20	11 (55.0)	9 (45.0)	62 (50-70)	52.0 (26.2-91.2)
University of Naples Federico II, Naples, Italy	11	6 (54.5)	5 (45.5)	65 (54-71)	42.0 (30.0-88.0)
Sapienza University of Rome, Rome, Italy	16	10 (62.5)	6 (37.5)	57 (50-68)	55.0 (30.5-110.8)
68 Medical centers in China^a^	499	309 (61.9)	190 (38.1)	57 (45-67)	27.5 (10.0-63.9)
6 Medical centers in Colombia^b^	7	6 (85.7)	1 (14.3)	47 (43-64)	33.0 (27.0-53.5)
Overall	1010	610 (60.4)	400 (39.6)	58 (46-68)	41.0 (14.8-84.9)

^a^ Chinese centers include Queen Mary Hospital, the University of Hong Kong, Hong Kong; Shanghai Tenth People’s Hospital, Shanghai; Shanghai General Hospital, Shanghai; Shanghai Sixth People’s Hospital, Shanghai; Shanghai Changzheng Hospital, Shanghai; Xiangya Hospital, Central South University, Changsha; The First Hospital of China Medical University, Shenyang; The Seventh Affiliated Hospital, Sun Yat-sen University, Shenzhen; Sun Yat-sen Memorial Hospital, Sun Yat-sen University, Guangzhou; The Sixth Affiliated Hospital, Sun Yat-sen University, Guangzhou; Sun Yat-sen University Cancer Center, Guangzhou; The First Affiliated Hospital, Sun Yat-sen University, Guangzhou; China-Japan Friendship Hospital, Beijing; The Affiliated Hospital of Inner Mongolia Medical University, Hohhot; Peking Union Medical College Hospital, Beijing; Peking University People’s Hospital, Beijing; Peking University Cancer Hospital, Beijing; Nanjing Drum Tower Hospital, Nanjing; Nanchong Central Hospital, Nanchong; Nanfang Hospital, Southern Medical University, Guangzhou; The First Hospital of Jilin University, Changchun; Jilin Cancer Hospital, Changchun; Harbin Medical University Cancer Hospital, Harbin; Jiaxing First Hospital, Jiaxing; Jiaxing Second Hospital, Jiaxing; West China Hospital, Sichuan University, Chengdu; Sichuan Provincial People’s Hospital, Chengdu; Zhongshan Hospital, Fudan University, Shanghai; Huashan Hospital, Fudan University, Shanghai; Fudan University Shanghai Cancer Center, Shanghai; Daqing Longnan Hospital, Daqing; The Second Hospital of Dalian Medical University, Dalian; Tianjin Medical University Cancer Institute and Hospital, Tianjin; Yijishan Hospital, Wannan Medical College, Wuhu; Anhui Provincial Hospital, Hefei; Anhui Provincial Cancer Hospital, Hefei; Baoying County People’s Hospital, Yangzhou; Qilu Hospital of Shandong University, Jinan; Shandong Cancer Hospital, Jinan; Shanxi Cancer Hospital, Taiyuan; Changzhou First People’s Hospital, Changzhou; Guangdong Provincial People’s Hospital, Guangzhou; Guangxi Medical University Cancer Hospital, Nanning; Xuzhou Central Hospital, Xuzhou; Union Hospital, Tongji Medical College, Huazhong University of Science and Technology, Wuhan; Tongji Hospital, Tongji Medical College, Huazhong University of Science and Technology, Wuhan; Renmin Hospital of Wuhan University, Wuhan; Jiangsu Province Hospital, Nanjing; Jiangsu Cancer Hospital, Nanjing; Henan Cancer Hospital, Zhengzhou; Taizhou Hospital of Traditional Chinese Medicine, Taizhou; The First Affiliated Hospital, Zhejiang University School of Medicine, Hangzhou; Sir Run Run Shaw Hospital, Zhejiang University School of Medicine, Hangzhou; Zhejiang Cancer Hospital, Hangzhou; The First Affiliated Hospital of Wenzhou Medical University, Wenzhou; Hunan Cancer Hospital, Changsha; Fujian Medical University Union Hospital, Fuzhou; Fujian Cancer Hospital, Fuzhou; Subei People’s Hospital, Yangzhou; The First Affiliated Hospital of Soochow University, Suzhou; Suzhou Municipal Hospital, Suzhou; Liaoning Cancer Hospital, Shenyang; The Affiliated Hospital of Zunyi Medical University, Zunyi; The First Affiliated Hospital of Zhengzhou University, Zhengzhou; Daping Hospital, Army Medical University, Chongqing; Changhai Hospital, Naval Medical University, Shanghai; The Affiliated Hospital of Qingdao University, Qingdao; Qinghai Fifth People’s Hospital, Xining.

^b^ Colombian centers include Centro de Tratamiento e Investigación sobre Cáncer Luis Carlos Sarmiento Angulo, Bogotá; Clínica Universitaria Colombia, Bogotá; Hospital Méderi, Bogotá; Instituto Nacional de Cancerología, Bogotá; Fundación Valle del Lili, Cali; and Clínica Colsanitas, Medellín.

#### US Surveillance, Epidemiology, and End Results Database

External validation of the proposed PDTC staging system was performed using the US Surveillance, Epidemiology, and End Results (SEER) database, with data accessed from the SEER 17 Registries Custom Data (November 2024 submission; released April 2025). Patients with PDTC diagnosed from 2004 to 2015 were selected using *International Classification of Diseases for Oncology, 3rd Edition* (*ICD*-*O*-*3*) codes C73.9 and 8337/3 to maximize specificity. These patients were filtered using inclusion and exclusion criteria consistent with the IATCCG cohort. AJCC/UICC TNM-8 and modified N stages were reclassified using SEER variables for tumor size, extension, lymph nodes, and distant metastases. As SEER includes deidentified public data, this component of the study was granted an exemption from institutional review board review.

### Outcomes

The primary end point of this study was overall survival (OS), which was defined as the time from initial surgery to death from any cause. Patients who were alive at the last follow-up were censored at the date of last known contact.

### Statistical Analysis

The proposed staging system was derived using data from the IATCCG cohort and externally validated in the SEER cohort through a 3-step workflow. Comprehensive methodological details are provided in the eMethods in Supplement 1.

Survival curves were generated using the Kaplan-Meier method and compared with the log-rank test to evaluate the differences in OS. Categorical variables were compared using Pearson χ^2^ test, and continuous variables were compared using *t* tests or the Mann-Whitney *U* tests, as appropriate. All statistical tests were 2-sided, and *P* < .05 was considered statistically significant. All analyses were performed using R software version 4.5.2 (The R Foundation). Data were analyzed from December 2025 to June 2026.

## Results

### Cohort Characteristics

The final IATCCG cohort included a total of 1010 patients (Table 1). In this cohort, 610 (60.4%) were female and 400 (39.6%) were male, and the median (IQR) age at diagnosis was 58 (46-68) years. At initial diagnosis, 219 patients (21.7%) presented with distant metastasis (M1), and 187 (18.5%) were classified as stage IV. Most patients (782 [77.4%]) underwent total thyroidectomy. During the follow-up period (median [IQR] of 41 [15-85] months), 341 deaths (33.8%) were recorded (eTable in Supplement 1).

The US SEER cohort included 170 patients; 85 (50.0%) were female, 85 (50.0%) were male, and the median (IQR) age at diagnosis was 61 (49-72) years. At presentation, 32 patients (18.8%) had M1 disease, and 33 (19.4%) were classified as having AJCC/UICC TNM-8 stage IV disease. Total thyroidectomy was performed in 153 patients (90.0%). Over a median (IQR) follow-up of 92 (42-154) months, 106 deaths (62.4%) occurred (eTable in Supplement 1).

### Modification of the N Category

Inspired by a previous work on medullary thyroid cancer,^13^ we reassessed the impact of LNM sites on PDTC prognosis to refine N staging. Although the AJCC/UICC TNM-8 merges N0 (pN0) and NX (cN0) in stage grouping for DTC and anaplastic thyroid cancer, this approach lacks validation in PDTC. Survival analysis in the IATCCG cohort showed comparable survival between the N0 and NX groups (5-year OS rates, 75.5% [95% CI, 67.2-82.0] vs 73.7% [95% CI, 68.3-78.4], respectively; *P* = .81) (eFigure 2 in Supplement 1). Consequently, we retained N0 and NX as a combined category (N0/NX) for further TNM stage grouping.

Systematic evaluation highlighted level VII LNM as a distinct aggressive phenotype. Analysis of dissemination patterns showed that 47 of 70 patients (67.1%) with level VII involvement harbored concurrent lateral neck metastases, while only 47 of 272 (17.3%) with lateral neck disease had spread to level VII, indicating level VII as a higher nodal burden (Figure 1A). Consistent with this, level VII LNM was associated with elevated rates of advanced T stage (T3-T4: 92.9% [65 of 70]) and distant metastasis (M1: 40.0% [28 of 70]) compared with none/no evidence, level VI, and lateral neck groups (Figure 1B). Furthermore, the R2 resection rate peaked at 22.9% (16 of 70) in the level VII group, markedly exceeding rates in the none/no evidence (4.6% [27 of 588]), level VI (5.5% [7 of 127]), and lateral neck (12.0% [27 of 225]) cohorts (Figure 1C). Crucially, level VII LNM portended the worst survival, with a 5-year OS of 45.2% (95% CI, 33.4-61.2). This was significantly inferior to the none/no evidence (74.4%; 95% CI, 70.4-78.7; log-rank *P* < .001), level VI (73.2%; 95% CI, 64.4-83.3; *P* < .001), and lateral neck (56.7%; 95% CI, 49.4-65.0; *P* = .04) groups (Figure 1D). Based on these disparities in dissemination, disease burden, resectability, and survival, we propose a modified N classification designating level VII LNM as a new N1c category within the existing AJCC framework.

**Figure 1.**
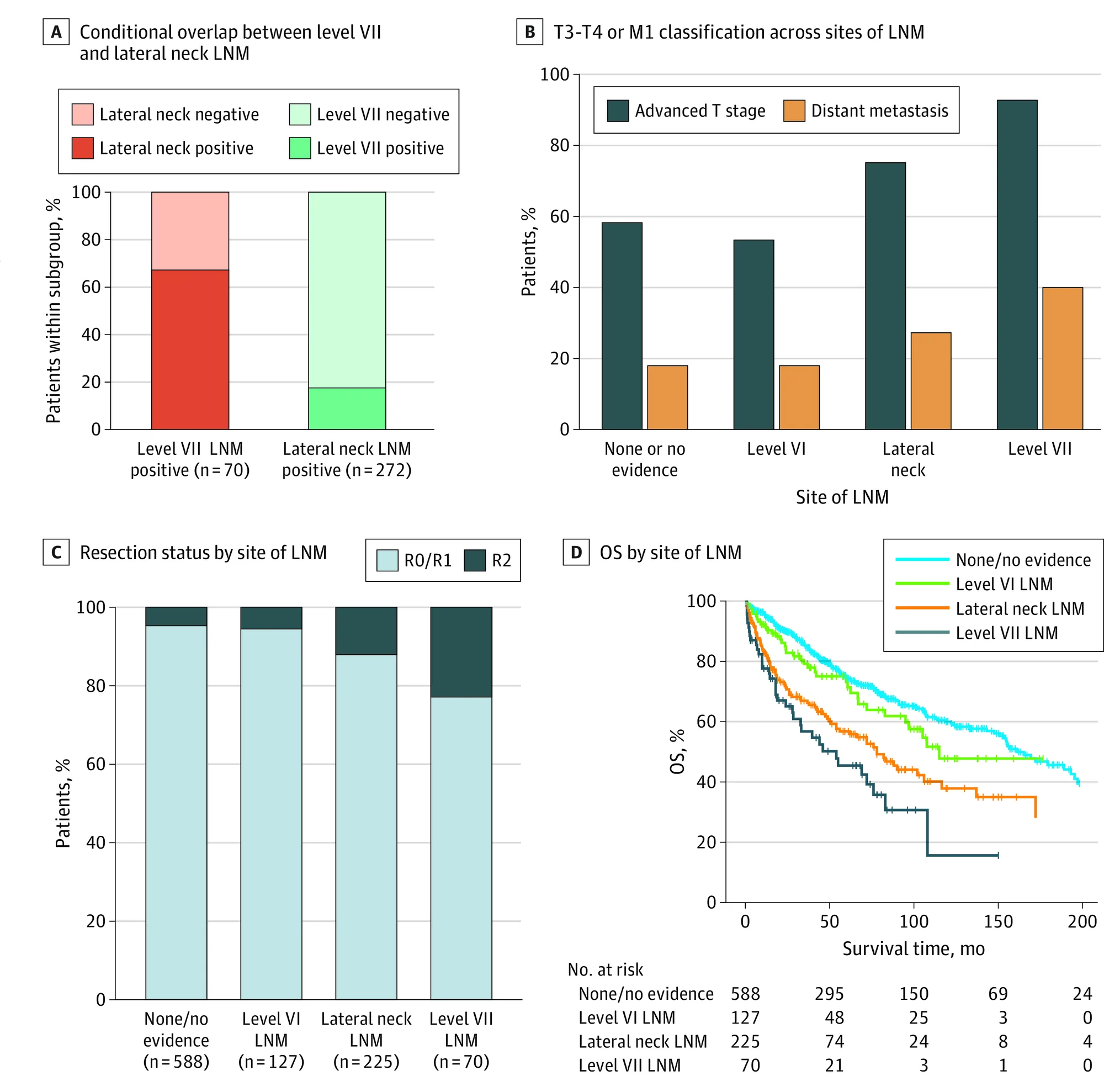
Clinicopathologic Features of Metastatic Nodal Sites in Poorly Differentiated Thyroid Cancer A, Stacked bar graph showing conditional overlap between level VII (upper mediastinal) lymph node metastasis (LNM) and lateral neck LNM within the International Aggressive Thyroid Cancer Collaborative Group cohort. B, Bar graph showing prevalence of advanced T stage (T3 to T4) and distant metastasis (M1) across nodal sites. Statistical significance was assessed via pairwise Fisher exact tests with Holm correction. Level VII LNM was associated with significantly higher rates of advanced T stage compared with the none/no evidence, level VI, and lateral neck groups and significantly higher rates of distant metastasis compared with the none/no evidence and level VI groups. C, Stacked bar graph showing distribution of surgical resection status (R0/R1 vs R2) across metastatic nodal sites. Level VII LNM was associated with higher R2 resection compared with the none/no evidence, level VI, and lateral neck groups. D, Kaplan-Meier curves for overall survival (OS) according to metastatic nodal sites.

### Derivation of the Proposed PDTC Staging System

Prior to staging development, we evaluated whether the 55-year age cutoff in AJCC/UICC TNM-8 for DTC remains applicable to PDTC. The core rationale behind this is that even with distant metastasis, patients with DTC younger than 55 years have a significantly better prognosis than older patients.^14^ However, survival analysis revealed that among patients with M1 disease in the IATCCG cohort, there was no significant prognostic difference between those older and younger than 55 years (eFigure 3 in Supplement 1), reflecting the different biology of this disease. Thus, this age threshold for DTC was discarded for our PDTC staging model.

As detailed in the eMethods in Supplement 1, an exhaustive enumeration of 16 738 T–modified N combinations was performed in 791 patients with M0 PDTC (201 deaths), each fitted into a multivariable Cox model adjusting for baseline covariates. We found that the AJCC/UICC TNM-8 staging yielded an Akaike information criterion of 4020.22, exceeding all enumerated configurations, highlighting substantial room for improvement over current standards. The optimal model (Akaike information criterion of 3969.81) defined the proposed stages I to IVA as follows: stage I, T1N0/NX/N1aM0 or T2N0/NXM0; stage II, T1N1b-N1cM0, T2N1a-N1bM0, or T3N0/NX/N1aM0; stage III, T2N1cM0, T3N1bM0, or T4aN0/NX/N1aM0; stage IVA, T3N1cM0, T4aN1b-N1cM0, or T4b any N M0; stage IVB, M1 disease (any T, any N, M1) (eMethods and eFigure 4 in Supplement 1; Table 2 and Box). Within the proposed system, all evaluable T–modified N combinations within a given M0 stage exhibited lower adjusted hazard ratios than those assigned to any higher stage (eFigure 5 in Supplement 1), whereas AJCC/UICC TNM-8 showed risk-ordering inconsistencies across stage groups (eFigure 5 in Supplement 1).

**Table 2.** TNM-8 Stage (DTC Criteria) and Proposed PDTC Stage Definitions

Category	TNM-8 staging (DTC criteria) definition	Proposed PDTC-specific staging definition
**T category**		
T1	Tumor ≤2 cm, confined to the thyroid	No change
T1a	Tumor ≤1 cm, confined to the thyroid	No change
T1b	Tumor >1-≤2 cm, confined to the thyroid	No change
T2	Tumor >2-≤4 cm, confined to the thyroid	No change
T3	Tumor >4 cm limited to the thyroid or gross extrathyroidal extension invading only strap muscles	No change
T3a	Tumor >4 cm limited to the thyroid	No change
T3b	Gross extrathyroidal extension invading only strap muscles (sternohyoid, sternothyroid, thyrohyoid, or omohyoid muscles) from a tumor of any size	No change
T4	Includes gross extrathyroidal extension into major neck structures	No change
T4a	Gross extrathyroidal extension invading subcutaneous soft tissues, larynx, trachea, esophagus, or recurrent laryngeal nerve from a tumor of any size	No change
T4b	Gross extrathyroidal extension invading prevertebral fascia or encasing carotid artery or mediastinal vessels from a tumor of any size	No change
**N category**		
NX	Regional lymph nodes cannot be assessed	No change
N0	No evidence of locoregional lymph node metastasis	No change
N1		
N1a	Metastasis to level VI or VII lymph nodes	Metastasis to level VI lymph nodes
N1b	Metastasis to level I-V or retropharyngeal lymph nodes	Metastasis to level I-V or retropharyngeal lymph nodes
N1c	NA	Metastasis to level VII lymph nodes
**M category**		
M0	No distant metastasis	No change
M1	Distant metastasis	No change

Abbreviations: DTC, differentiated thyroid cancer; NA, not applicable; PDTC, poorly differentiated thyroid cancer; TNM-8, eighth version of the American Joint Committee on Cancer and Union for International Cancer Control TNM staging system.

Box. **TNM-8 Stage (DTC Criteria) and Proposed PDTC Staging**

**Stage I**

TNM-8 Staging (DTC Criteria)
  Age <55 y: Any T, any N, M0
  Age ≥55 y: T1/T2, N0/Nx, M0
Proposed PDTC-Specific Staging
  T1N0/NX/N1aM0 or T2N0/NXM0

**Stage II**

TNM-8 Staging (DTC Criteria)
  Age <55 y: Any T, any N, M1;
  Age ≥55 y: T1/T2N1M0 or T3, any N, M0
Proposed PDTC-Specific Staging
  T1N1b/N1cM0, T2N1a/N1bM0, or T3N0/NX/N1aM0

**Stage III**

TNM-8 Staging (DTC Criteria)
  Age ≥55 y: T4a, any N, M0
Proposed PDTC-Specific Staging
  T2N1cM0, T3N1bM0, or T4aN0/NX/N1aM0

**Stage IVA**

TNM-8 Staging (DTC Criteria)
  Age ≥55 y: T4b, any N, M0
Proposed PDTC-Specific Staging
  T3N1cM0, T4aN1b/N1cM0; or T4b, any N, M0

**Stage IVB**

TNM-8 Staging (DTC Criteria)
  Age ≥55 y: Any T, any N, M1
Proposed PDTC-Specific Staging
  Any T, any N, M1

Abbreviations: DTC, differentiated thyroid cancer; PDTC, poorly differentiated thyroid cancer; TNM-8, eighth version of the American Joint Committee on Cancer and Union for International Cancer Control TNM staging system.

Overall, 370 patients (36.6%) in the IATCCG cohort were reclassified by the proposed PDTC staging. Compared with the AJCC/UICC TNM-8 (DTC criteria), the proposed PDTC-specific system decreased the proportion of patients with stage I disease from 44.4% (448 of 1010) to 24.6% (248 of 1010), while the proportion of patients with stage IV disease increased from 18.5% (187 of 1010) to 32.4% (327 of 1010). In summary, the new system reduced the concentration of patients in early stages and reallocated those with metastatic or high-risk disease to appropriate advanced stages (eFigure 4 in Supplement 1).

### Prognostic Performance and External Validation

In the IATCCG cohort, AJCC/UICC TNM-8 (DTC criteria) yielded 5-year OS rates of 85.1% (95% CI, 80.7-88.6) for stage I, 66.6% (95% CI, 59.9-72.5) for stage II, 58.0% (95% CI, 44.6-69.2) for stage III, 38.5% (95% CI, 12.1-64.9) for stage IVA, and 36.7% (95% CI, 28.1-45.3) for stage IVB, failing to stratify patients adequately between stages II and III or between stages IVA and IVB (Figure 2A and B). In contrast, the proposed PDTC-specific system yielded a more clearly delineated, progressive decline in OS across stages, with 5-year OS of 89.2% (95% CI, 83.8-92.9) for stage I, 78.8% (95% CI, 72.4-83.8) for stage II, 68.3% (95% CI, 57.6-76.8) for stage III, 53.8% (95% CI, 42.0-64.2) for stage IVA, and 38.2% (95% CI, 30.8-45.6) for stage IVB (Figure 2C and D). Furthermore, subgroup analyses confirmed that among the 2 continents (Asia and Europe) with adequate patient enrollment (more than 200 cases), the proposed staging also revealed clearer survival discrimination than AJCC/UICC TNM-8 (eFigure 6 in Supplement 1).

**Figure 2.**
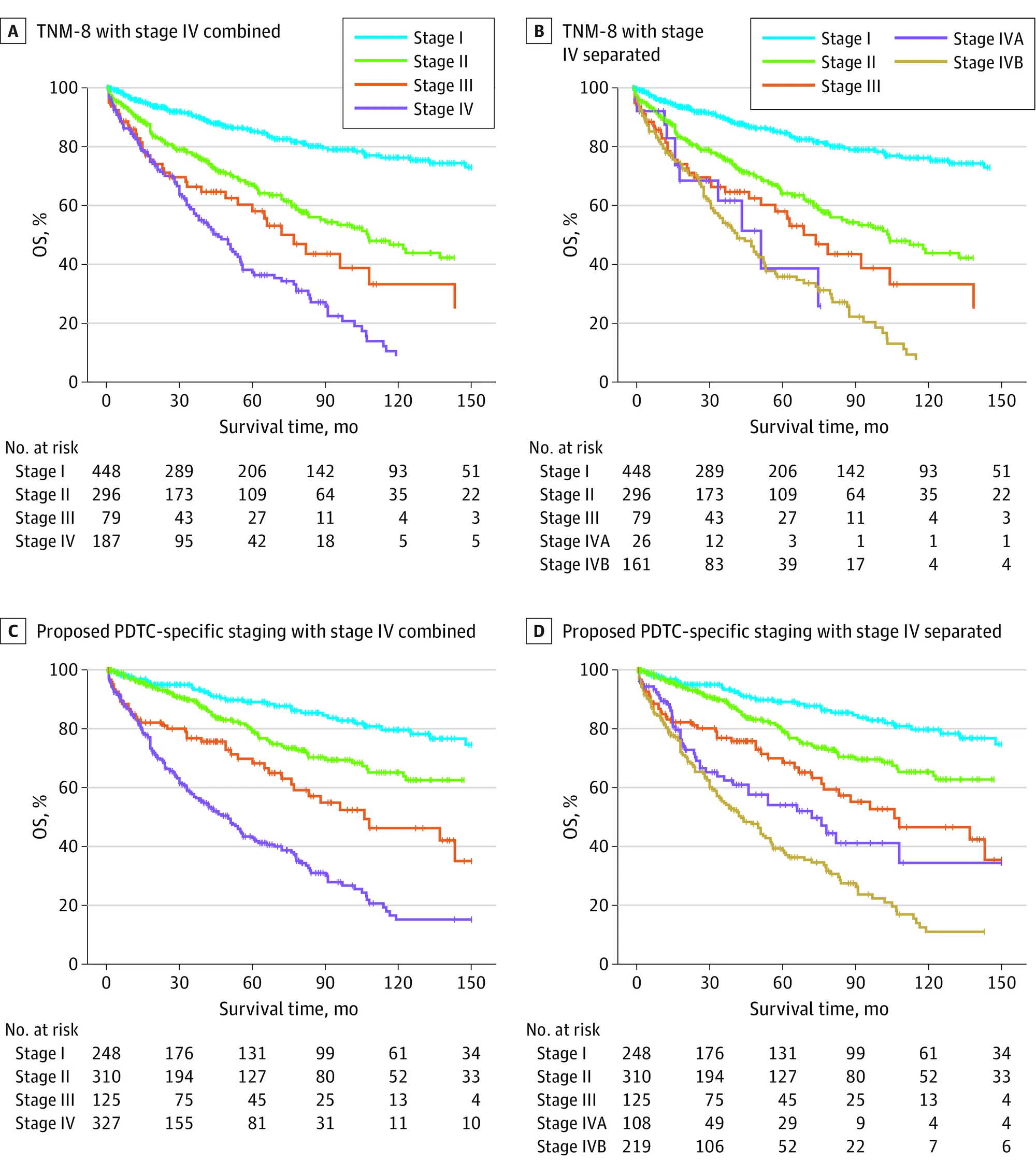
Kaplan-Meier Curves for Overall Survival (OS) by Staging System in the International Aggressive Thyroid Cancer Collaborative Group Cohort A and B, Kaplan-Meier curves for OS according to the eighth version of the American Joint Committee on Cancer and Union for International Cancer Control TNM staging system (TNM-8; differentiated thyroid cancer criteria), with stage IVA and IVB combined and with stage IVA and IVB separated. C and D, Kaplan-Meier curves for OS according to the proposed poorly differentiated thyroid cancer (PDTC)–specific staging system, with stage IVA and IVB combined and with stage IVA and IVB separated.

Similarly, in the US SEER cohort, AJCC/UICC TNM-8 (DTC criteria) showed insufficient OS separation, with 5-year rates of 88.5% (95% CI, 78.3-94.1) for stage I, 60.3% (95% CI, 45.5-72.3) for stage II, 61.5% (95% CI, 30.8-81.8) for stage III, 22.2% (95% CI, 3.4-51.3) for stage IVA, and 45.8% (95% CI, 25.6-64.0) for stage IVB and persistent overlap between stages II and III and between stages IVA and IVB (Figure 3A and B). In contrast, the proposed PDTC-specific system reproduced a more ordered survival gradient, supporting its generalizability. With stage IV subdivided, 5-year OS declined progressively from 97.1% (95% CI, 80.9-99.6) for stage I to 76.8% (95% CI, 64.5-85.3) for stage II, 54.5% (95% CI, 32.1-72.4) for stage III, 46.2% (95% CI, 19.2-69.6) for stage IVA, and 37.5% (95% CI, 21.3-53.7) for stage IVB, confirming robust prognostic stratification in this external validation (Figure 3C and D).

**Figure 3.**
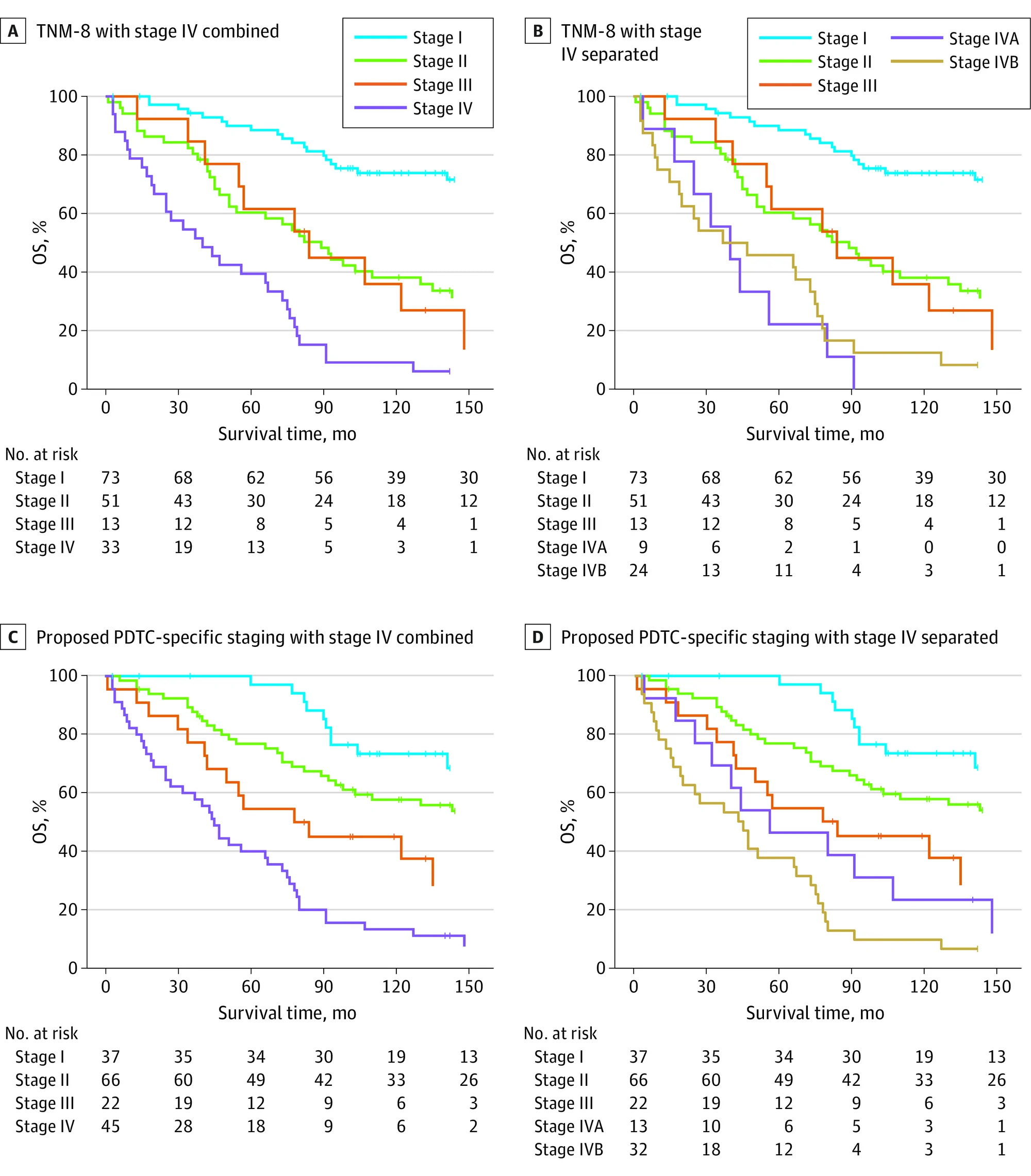
Kaplan-Meier Curves for Overall Survival (OS) by Staging System in the US Surveillance, Epidemiology, and End Results Cohort A and B, Kaplan-Meier curves for OS according to the eighth version of the American Joint Committee on Cancer and Union for International Cancer Control TNM staging system (TNM-8; differentiated thyroid cancer criteria), with stage IVA and IVB combined and with stage IVA and IVB separated. C and D, Kaplan-Meier curves for OS according to the proposed poorly differentiated thyroid cancer (PDTC)–specific staging system, with stage IVA and IVB combined and with stage IVA and IVB separated.

Multivariable Cox models adjusting for all prespecified relevant baseline covariates demonstrated a more consistent monotonic stagewise mortality gradient for the proposed staging system compared with the AJCC/UICC TNM-8 classification in both cohorts, specifically by resolving the previously limited discriminatory capacity between stages II and III (eFigure 7 in Supplement 1). Consistently, the proposed PDTC-specific staging system showed higher C statistic values than AJCC/UICC TNM-8 in both the IATCCG cohort (0.74 [95% CI, 0.71-0.76] vs 0.71 [95% CI, 0.68-0.74]; difference, 0.03; 95% CI, 0.01-0.04) and the US SEER cohort (0.76 [95% CI, 0.71-0.81] vs 0.74 [95% CI, 0.68-0.79]; difference, 0.02; 95% CI, −0.01 to 0.06).

Furthermore, sensitivity analyses demonstrated that sequentially excluding any major contributing center (50 or more patients; 10 or more events), the 2 largest continents (Asia and Europe), or the largest contributing country (China) yielded consistent results; event rates and adjusted hazard ratios for stages I to IV maintained a monotonic increase, and the C statistic values remained superior to that of AJCC/UICC TNM-8 system (eFigures 8 and 9 in Supplement 1). Similarly, in subgroups defined by year of diagnosis (before 2015, 2015 to 2019, and 2020 onward), the survival curves for stages I to IV of the proposed staging system were also basically separated (eFigure 10 in Supplement 1). These findings confirm that the robust performance of the proposed staging system is not attributable to any single institution.

## Discussion

Despite the iterative refinements of conventional TNM staging systems, frameworks for PDTC remained stagnant. To address this limitation, we initiated a global collaborative endeavor, integrating data across 14 countries to build the largest real-world PDTC cohort available. This robust infrastructure enabled us to develop and externally validate a disease-specific TNM staging model for this rare disease.

In our proposed PDTC-specific staging system, we made 2 major modifications compared with DTC criteria. First, we eliminated the 55-year age cutoff. In the DTC criteria, patients younger than 55 years have an overall favorable prognosis even with distant metastasis (stage II), which is significantly better than in those older than 55 years (stage IVB), likely due to the higher propensity for dedifferentiation in older patients.^10^ However, our data from the IATCCG cohort confirm that survival outcomes for patients with metastatic PDTC do not differ significantly by this age cutoff. This is likely because PDTC itself has already undergone dedifferentiation and therefore may offer no comparable grace period for younger patients. Second, similar to a prior study of medullary thyroid cancer staging,^13^ we re-evaluated the prognostic significance of level VII metastasis in PDTC. In AJCC/UICC TNM-8, level VII metastasis was downgraded from N1b in TNM-7 to N1a, grouping it with level VI as the mildest form of lymphadenopathy due to the anatomical continuity of the 2 regions.^10^ However, this classification proved inadequate for PDTC according to our data. In PDTC, level VII LNM signifies a heavier disease burden and, coupled with sternal obstruction and proximity to vital mediastinal vessels, results in significantly lower complete resection rates, correlating with worse survival. Thus, we propose a modified N classification by upgrading level VII LNM to a new N1c category.

The prognostic impact of level VII LNM in PDTC underscores the need for precise nodal delineation in operative and pathology reports. Since level VII involvement provides prognostic information beyond level VI status, separate reporting of these nodal stations may refine our interpretation of staging, recurrence patterns, and the extent of mediastinal disease. Level VII should be anatomically defined as the upper mediastinal compartment bounded by the suprasternal notch and the innominate artery. Such standardized documentation is crucial for guiding future refinements in thyroid cancer staging.

Early attempts have been made to stage PDTC using SEER data, but these endeavors were compromised by inconsistent pathological definitions.^12^ Sun et al^12^ used *ICD*-*O*-*3* code 8020/3, a designation that predominantly captures anaplastic thyroid cancer rather than PDTC. Guo et al^17^ adopted a more inclusive strategy by combining grade III with a broad spectrum of *ICD*-*O*-*3* codes (8050/3, 8260/3, 8290/3, 8330/3, 8331/3, 8332/3, 8335/3, 8337/3, 8340/3, 8341/3, 8342/3, 8343/3, and 8344/3). However, this list was heavily contaminated with DTC and their related variants; only 8337/3 (insular carcinoma) aligns with PDTC pathology. Consequently, inclusion of these lower-risk phenotypes would inevitably dilute the prognostic signal and introduce substantial selection bias.

Unlike standardized management of DTC, treatment of PDTC remains highly variable due to the rarity of the disease. Controversies persist regarding the optimal extent of thyroidectomy, the role of prophylactic neck dissection, and the efficacy of adjuvant therapies, including radioactive iodine and external beam radiation therapy, while evidence for systemic therapies is even scarcer.^7^ Although total thyroidectomy with resection of all gross disease can theoretically improve local control,^18^ advanced presentation often precludes ideal extirpation, resulting in palliative debulking for many cases in clinical practice. Given this therapeutic heterogeneity, we included patients undergoing palliative resection, subtotal thyroidectomy, and those without prophylactic neck dissection, as these practices align with guidelines and are common in clinical practice. This pragmatic approach ensures our staging system mirrors real-world complexity.

The IATCCG alliance formed the basis of this study by assembling the world’s largest PDTC cohort from 14 countries across 6 continents. This global representation is essential for developing a universally applicable staging system aligned with AJCC/UICC objectives. However, given that the IATCCG cohort originates solely from high-volume referral centers, the resulting model carries the risk of limited generalizability to community or nonspecialized clinical settings. We therefore validated the model against the SEER database, reflecting broader US health care settings. Patients in the SEER cohort had a lower proportion of N1 disease than patients in the IATCCG cohort (eTable in Supplement 1). This difference may reflect differences in referral patterns and nodal assessment between the cohorts. Crucially, the model retained robust prognostic discrimination in SEER, underscoring its stability across diverse clinical settings and demonstrating its value beyond specialized academic centers.

Integrating molecular features into conventional staging systems has emerged as a promising direction to refine risk stratification in thyroid cancer. Recent large-scale international studies have demonstrated that incorporating *BRAF* and *TERT* promoter variants into the AJCC classification significantly improves mortality risk prediction.^20^ For PDTC specifically, recurrent alterations in *TP53*, *TERT* promoter, *BRAF*, and *RAS* have been identified as potential prognostic markers or therapeutic targets.^7^ Notably, *BRAF* V600E variants render PDTC amenable to treatment with BRAF/MEK inhibitors, which have shown efficacy in anaplastic thyroid cancer and are being explored in the PDTC setting,^22^ while *RAS*-variant tumors may benefit from mitogen-activated protein kinase pathway blockade or multikinase inhibitors, although clinical data specific to PDTC remain limited. Although molecular data were not fully available in our multicenter cohort, future studies incorporating comprehensive genomic profiling may further refine the proposed staging framework and inform personalized treatment strategies.

### Limitations

This study has limitations. First, limiting the cohort to surgically managed patients may underrepresent inoperable advanced cases. However, PDTC lacks reliable biomarkers and relies solely on morphological criteria, precluding definitive diagnosis and exclusion of anaplastic foci via cytology or core biopsies. Thus, surgery remains essential not only for treatment but also for diagnosis. Second, although we adhered to the standardized PDTC diagnostic code (*ICD*-*O*-*3* code 8337/3) for SEER data, its registry-based nature precluded pathological review. Third, there remains no standard of care for adjuvant therapy, and therapeutic strategies varied substantially across institutions, with patients with advanced disease often exposed to multiple lines of therapy. Given this heterogeneity and classification challenges, adjuvant therapies were omitted from the baseline covariate set for multivariable adjustment. Finally, the latest World Health Organization classification introduces high-grade DTC, an aggressive entity biologically intermediate between DTC and anaplastic thyroid cancer. While high-grade DTC is thought to behave similarly to PDTC,^9^ it is defined by different pathological criteria, rather than the Turin criteria. Whether the proposed PDTC staging system is applicable to high-grade DTC warrants independent validation in future studies based on rigorous pathological review.

## Conclusions

In conclusion, via a major global collaboration, this study establishes a PDTC-specific TNM staging system aligning with its aggressive biology. It offers superior prognostic stratification and a practical framework for global clinical management. These findings underscore that global collaboration is essential for developing tailored staging systems for rare, aggressive cancers, ensuring the TNM system’s continued global relevance.

## Notes

Supplement 1.
eMethods.
**eTable.** Baseline Clinicopathologic Characteristics of the IATCCG and US SEER Cohorts **eFigure 1. **Patient Selection Flow Diagram for the IATCCG Cohort **eFigure 2.** Kaplan-Meier Curves for Overall Survival Comparing N0 (pN0) and NX (cN0) Patients in the IATCCG Cohort **eFigure 3.** Kaplan-Meier Estimates of Overall Survival for Patients Aged <55 Years and ≥55 Years With M1 Disease in the IATCCG Cohort **eFigure 4.** Exhaustive Enumeration and Stage Redistribution for the Proposed PDTC-Specific Staging System **eFigure 5.** Comparison of Proposed PDTC-Specific and AJCC/UICC TNM-8 Stage Grouping Matrices **eFigure 6.** Subgroup Analysis of Overall Survival Stratification in Asia and Europe **eFigure 7.** Adjusted HRs and 95% CIs by Staging System **eFigure 8.** Leave-1-Center-Out Sensitivity Analysis Comparing the Proposed Staging System With AJCC/UICC TNM-8 in the IATCCG Cohort **eFigure 9.** Geographic Exclusion Sensitivity Analysis **eFigure 10.** Kaplan-Meier Overall Survival Curves Stratified by Calendar Year of Diagnosis

Supplement 2. Group Information. International Aggressive Thyroid Cancer Collaborative Group

Supplement 3. Data Sharing Statement
