# Supplementary material for: Global Proposal for the TNM Staging System for Poorly Differentiated Thyroid Cancer
Source: JAMA Oncol. 2026 Nov 6:e264705. Online ahead of print. doi: 10.1001/jamaoncol.2026.4705 (PMC13744029; doi:10.1001/jamaoncol.2026.4705)
Supplement: Supplementary file 1 [file jamaoncol-e264705-s001.pdf]

Supplemental Online Content

Shi X, Liu Q, Scholfield D, et al. Global proposal for the TNM staging system for poorly differentiated thyroid cancer. *JAMA Oncol*. Published online November 6, 2026. doi:10.1001/jamaoncol.2026.4705

eMethods.

eTable. Baseline Clinicopathologic Characteristics of the IATCCG and US SEER Cohorts

eFigure 1. Patient Selection Flow Diagram for the IATCCG Cohort

eFigure 2. Kaplan-Meier Curves for Overall Survival Comparing N0 (pN0) and NX (cN0) Patients in the IATCCG Cohort

eFigure 3. Kaplan-Meier Estimates of Overall Survival for Patients Aged <55 Years and ≥55 Years With M1 Disease in the IATCCG Cohort

eFigure 4. Exhaustive Enumeration and Stage Redistribution for the Proposed PDTC-Specific Staging System

eFigure 5. Comparison of Proposed PDTC-Specific and AJCC/UICC TNM-8 Stage Grouping Matrices

eFigure 6. Subgroup Analysis of Overall Survival Stratification in Asia and Europe

eFigure 7. Adjusted HRs and 95% CIs by Staging System

eFigure 8. Leave-1-Center-Out Sensitivity Analysis Comparing the Proposed Staging System With AJCC/UICC TNM-8 in the IATCCG Cohort

eFigure 9. Geographic Exclusion Sensitivity Analysis

eFigure 10. Kaplan-Meier Overall Survival Curves Stratified by Calendar Year of Diagnosis

This supplementary material has been provided by the authors to give readers additional information about their work.

© 2026 Shi X et al. *JAMA Oncology*.

eMethods.

Detailed methodologies for pathological verification, patient exclusion, and TNM classification in the IATCCG cohort

To ensure diagnostic rigor, all PDTC cases strictly conformed to the Turin criteria as endorsed by the 5th edition of the WHO Classification.[1,2] Recognizing the historical absence of standardized diagnostic criteria,[1,3] pathological reassessment according to the Turin criteria was mandatory to verify eligibility. This process encompassed 80 historic diagnoses established prior to the formal adoption of the Turin criteria. While gold-standard re-review of histological slides was conducted whenever archival material was accessible, a subset of cases lacked available slides. In these instances, only tumors with comprehensive pathological reports explicitly documenting conformity to the Turin criteria were deemed eligible for inclusion. Furthermore, any tumor exhibiting anaplastic components was rigorously excluded, as the presence of such features is known to override the biological behavior and prognosis inherent to PDTC.

Initially, 1,098 patients diagnosed with PDTC were identified. Patients were subsequently excluded for the following reasons: (1) pathologic review failed to confirm the PDTC diagnosis according to the Turin criteria or revealed anaplastic components (n=35); (2) receipt of neoadjuvant therapy (n=21); (3) missing or indeterminate staging data (n=12; specifically regarding tumor size, extrathyroidal extension [ETE], or location of LNM); and (4) complete loss to follow-up or unknown survival status and time (n=20) **(eFigure 1 in the Supplement)**.

Baseline characteristics, including demographics, tumor features, surgical treatment details (surgery type and resection status), and follow-up data were recorded for all eligible patients. All study variables were standardized across participating institutions using a unified data dictionary, with central review to ensure consistency. T categorization was determined by tumor size and ETE per the AJCC 8th edition criteria. N staging was assigned based on the documented locations of LNM in surgical pathology reports. For unresectable nodal disease, staging relied on biopsy findings or a consensus evaluation by experienced radiologists interpreting preoperative imaging. Involvement of Level VII (anatomically bounded by the suprasternal notch and the innominate artery) was determined primarily through pathological nodal mapping, supplemented by operative notes and, when available, re-review of preoperative imaging, particularly in cases where differentiation from Level VI involvement was challenging.

Of note, unlike MTC's staging framework, which exclusively includes patients with clearly defined N categories, AJCC/UICC TNM-8 staging for DTC and ATC incorporates NX patients (cN0 without prophylactic neck dissection).[4]This aligns with clinical practice: prophylactic neck dissection is not universally required for early-stage DTC, and many patients with ATC undergo only palliative resection. Likewise, PDTC management is complicated by the low sensitivity of fine-needle biopsy for definitive preoperative diagnosis and persistent debate over prophylactic dissection in cN0 (NX) disease, often leading to surgery limited to tumor resection. Consequently, to adhere to AJCC standards and ensure clinical applicability, this PDTC study includes NX patients in its analytical cohort.

© 2026 Shi X et al. *JAMA Oncology*.

Comprehensive methodological details for the three-step workflow to derive and validate the proposed PDTC-specific staging system

1. **Step 1:** We then defined the candidate space for stage-group refinement. Metastatic (M1) disease was prespecified as stage IVB. For nonmetastatic (M0) disease, the number of stage groups was prespecified as four, corresponding to stages I, II, III and IVA, thereby preserving the same number of nonmetastatic stage categories used in AJCC/UICC TNM-8 (DTC criteria). Under this framework, all admissible mappings of T1–T4b × modified N combinations to the four nonmetastatic stage groups were enumerated under clinical monotonicity constraints. Stage assignments were constrained to be nondecreasing with increasing T category within each modified N category and with increasing modified N category within each T category. Candidate schemes were retained only if stages I, II, III, and IVA each included at least one patient. A total of 16,738 candidate staging schemas satisfied these constraints and were retained for subsequent model evaluation.
2. **Step 2:** Each admissible candidate scheme was mapped back to the patient level and included into a multivariable Cox proportional hazards model together with the prespecified baseline covariates, including age, sex, geographic region, extent of surgery and resection status. Akaike information criterion (AIC) was compared across all candidate Cox models, with a lower value indicating better relative model fit after accounting for model complexity. The candidate scheme with the lowest AIC was selected as the proposed staging schema.
3. **Step 3:** We then evaluated the discriminatory ability of the proposed staging system regarding OS in the IATCCG cohort using Kaplan-Meier estimates and the log-rank test, and externally validated its performance in the SEER cohort. Patients in each cohort were restaged according to both AJCC/UICC TNM-8 and the proposed staging system. We then assessed the monotonic progression of adjusted hazard ratios (aHRs) across Stages I-IV for both systems using multivariable Cox models adjusting for baseline covariates. The discriminative performance of the proposed staging system relative to AJCC/UICC TNM-8 was compared using Harrell's C-index derived from multivariable Cox models, with differences estimated by paired bootstrap resampling with 5,000 iterations. To account for the multi-institutional design, sensitivity analyses were performed by sequentially excluding centers meeting the thresholds of n ≥ 50 and events ≥ 10, thereby ruling out potential single-site bias. Additional sensitivity analyses evaluated model performance after separately excluding Asia, Europe, or China and across calendar-year-of-diagnosis groups (before 2015, 2015–2019, and 2020 onward).

References

1. Volante M, Collini P, Nikiforov YE, et al. Poorly differentiated thyroid carcinoma: the Turin proposal for the use of uniform diagnostic criteria and an algorithmic diagnostic approach. Am J Surg Pathol. 2007;31(8):1256-1264.
2. WHO Classification of Tumours Editorial Board: Endocrine and Neuroendocrine Tumours (ed 5th). Lyon (France), International Agency for Research on Cancer, 2025.
3. Tallini G. Poorly differentiated thyroid carcinoma. Are we there yet? Endocr Pathol.

© 2026 Shi X et al. *JAMA Oncology*.

2011;22(4):190-194.
4. Amin MB, Edge SB, Greene FL, Byrd DR, Brookland RK, Washington MK, et al., eds. AJCC Cancer Staging Manual. 8th ed. New York, NY: Springer; 2017.

© 2026 Shi X et al. *JAMA Oncology*.

eTable. Baseline Clinicopathologic Characteristics of the IATCCG and US SEER Cohorts

Characteristic	IATCCG Cohort, N (%)	US SEER cohort, N (%)	P value
Total	1,010	170	
Age, median (IQR)	58 (46–68)	61 (49–72)	.016
Duration of follow-up, months, median (IQR)	41 (15–85)	92 (42–154)	<.001
**Sex**			.011
Female	610 (60.4)	85 (50.0)	
Male	400 (39.6)	85 (50.0)	
**T category**			<.001
T1	152 (15.0)	6 (3.5)	
T2	213 (21.1)	37 (21.8)	
T3	432 (42.8)	91 (53.5)	
T4a	156 (15.4)	19 (11.2)	
T4b	57 (5.6)	17 (10.0)	
**N category**			.002
NX	382 (37.8)	77 (45.3)	
N0	206 (20.4)	48 (28.2)	
N1a	150 (14.9)	15 (8.8)	
N1b	272 (26.9)	30 (17.6)	
**M category**			.399
M0	791 (78.3)	138 (81.2)	
M1	219 (21.7)	32 (18.8)	
**AJCC/UICC TNM-8 Stage (DTC Criteria)**			.402
Stage I	448 (44.4)	73 (42.9)	
Stage II	296 (29.3)	51 (30.0)	
Stage III	79 (7.8)	13 (7.6)	
Stage IVA	26 (2.6)	9 (5.3)	
Stage IVB	161 (15.9)	24 (14.1)	
**Proposed PDTC-specific Stage**			.249
Stage I	248 (24.6)	37 (21.8)	
Stage II	310 (30.7)	66 (38.8)	
Stage III	125 (12.4)	22 (12.9)	

© 2026 Shi X et al. *JAMA Oncology*.

Characteristic	IATCCG Cohort, N (%)	US SEER cohort, N (%)	P value
Stage IVA	108 (10.7)	13 (7.6)	
Stage IVB	219 (21.7)	32 (18.8)	
**Geographic Region**			—
Asia	582 (57.6)	-	
Europe	215 (21.3)	-	
Oceania	92 (9.1)	-	
North America	87 (8.6)	170 (100)	
Africa	23 (2.3)	-	
South America	11 (1.1)	-	
**Extent of surgery**			<.001
Non-total thyroidectomy	228 (22.6)	17 (10.0)	
Total thyroidectomy	782 (77.4)	153 (90.0)	
**Resection status**			—
R0/R1	933 (92.4)	-	
R2	77 (7.6)	-	
**Vital status**			<.001
Alive	669 (66.2)	64 (37.6)	
Dead	341 (33.8)	106 (62.4)	

© 2026 Shi X et al. *JAMA Oncology*.

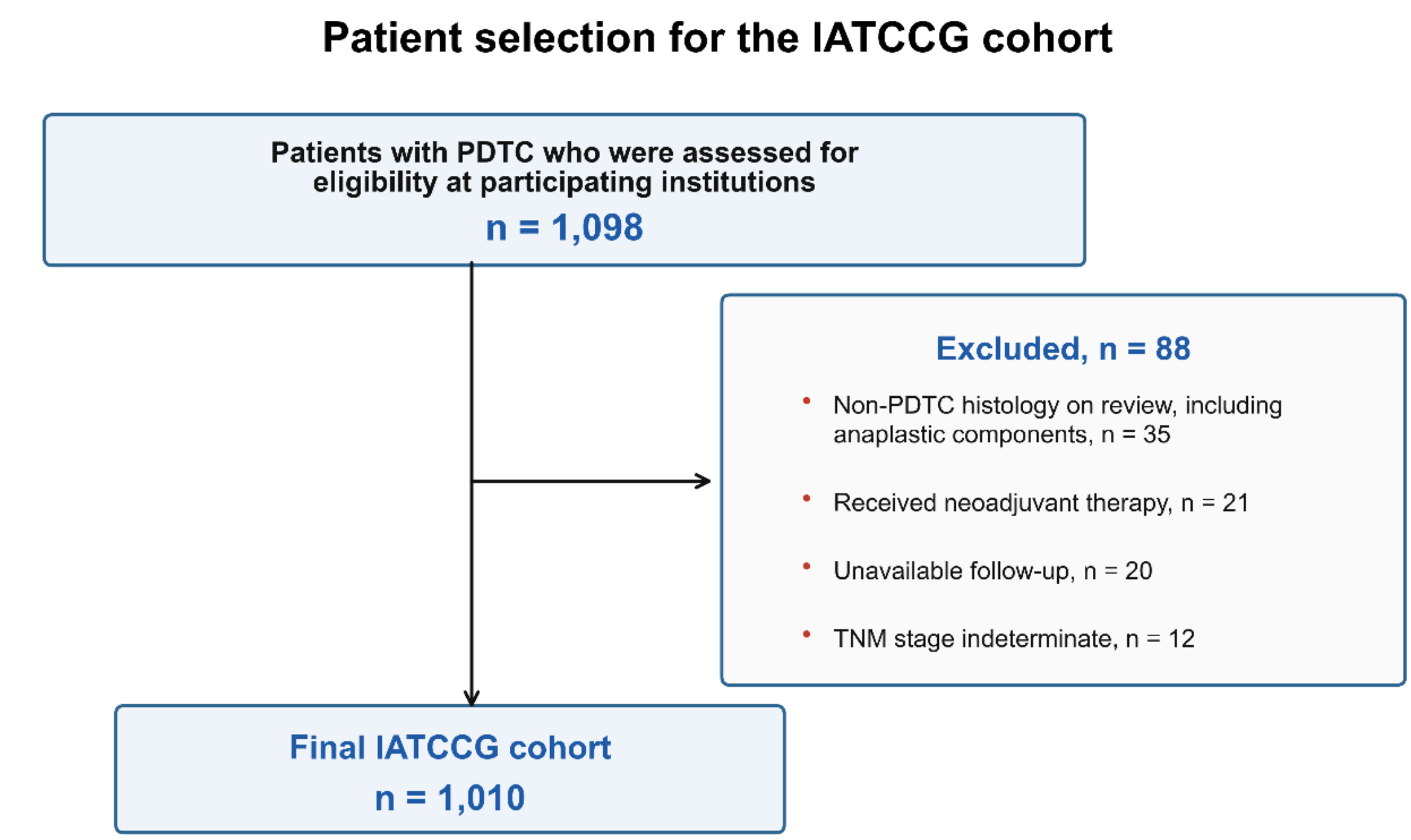

eFigure 1. Patient Selection Flow Diagram for the IATCCG Cohort

© 2026 Shi X et al. *JAMA Oncology*.

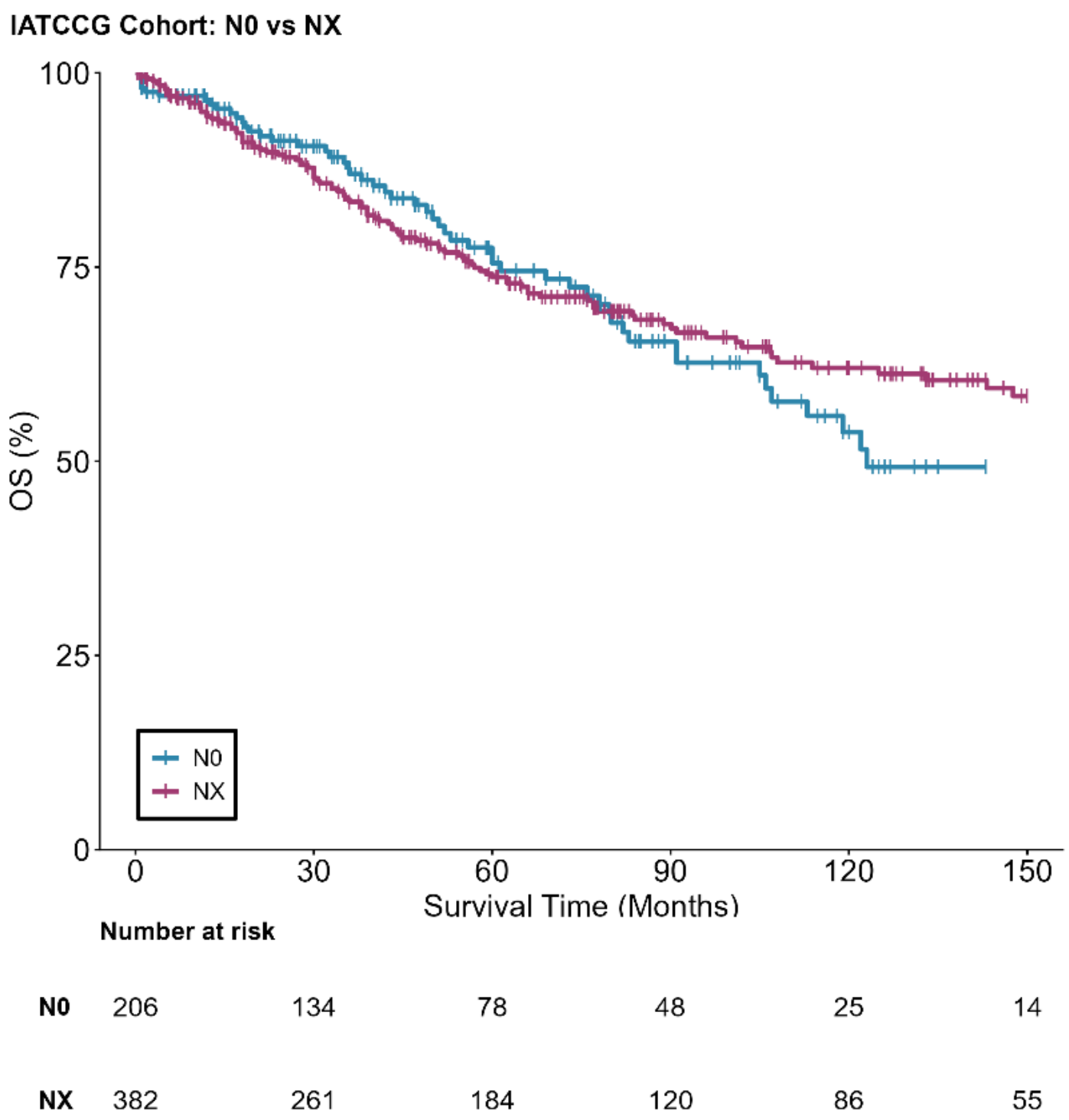

eFigure 2. Kaplan-Meier Curves for Overall Survival Comparing N0 (pN0) and NX (cN0) Patients in the IATCCG Cohort

NX (cN0) denotes patients without pathologic nodal assessment but clinically assessed as node-negative.

© 2026 Shi X et al. *JAMA Oncology*.

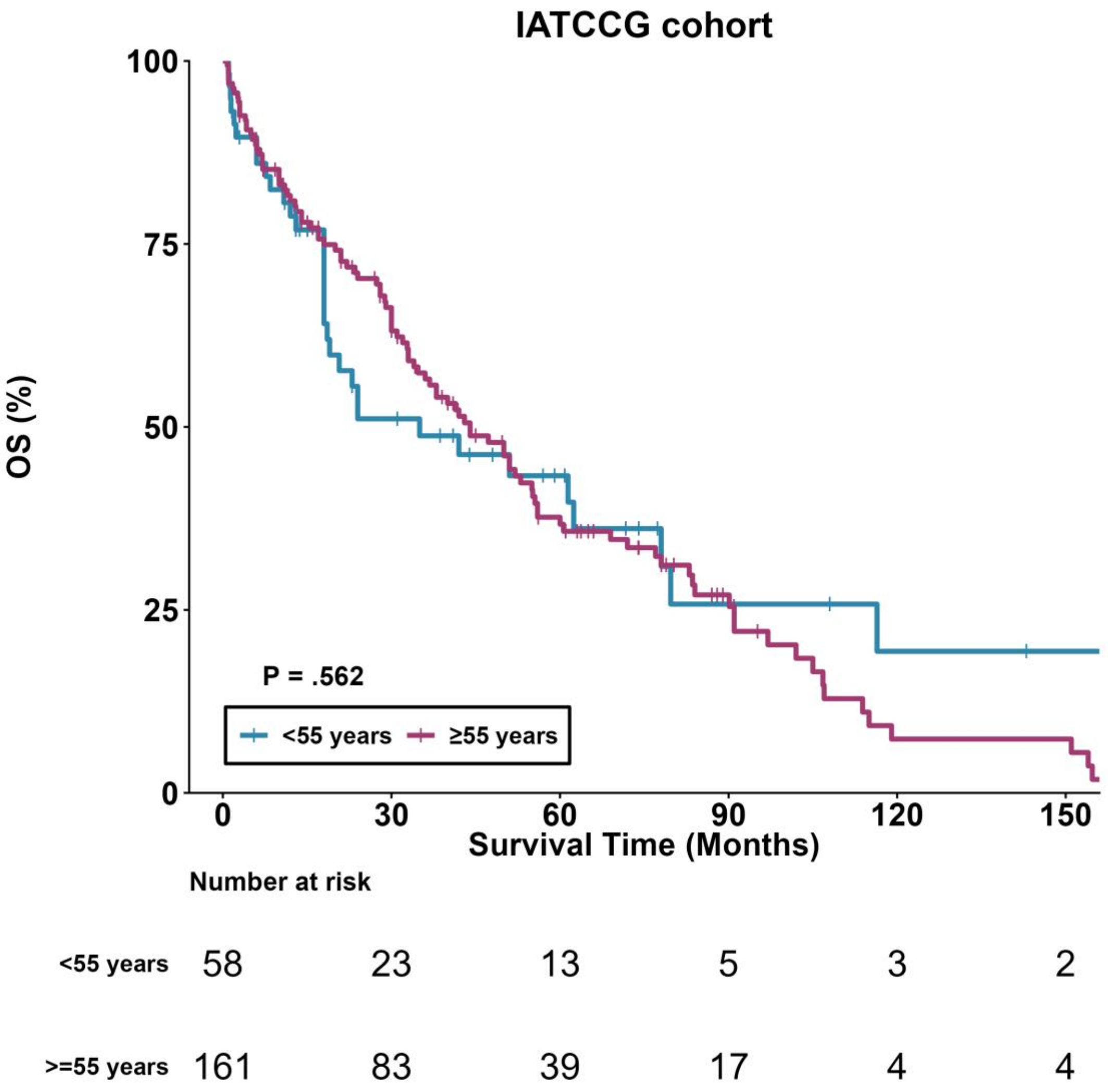

eFigure 3. Kaplan-Meier Estimates of Overall Survival for Patients Aged <55 Years and ≥55 Years With M1 Disease in the IATCCG Cohort

P values were calculated using the log-rank test.

© 2026 Shi X et al. *JAMA Oncology*.

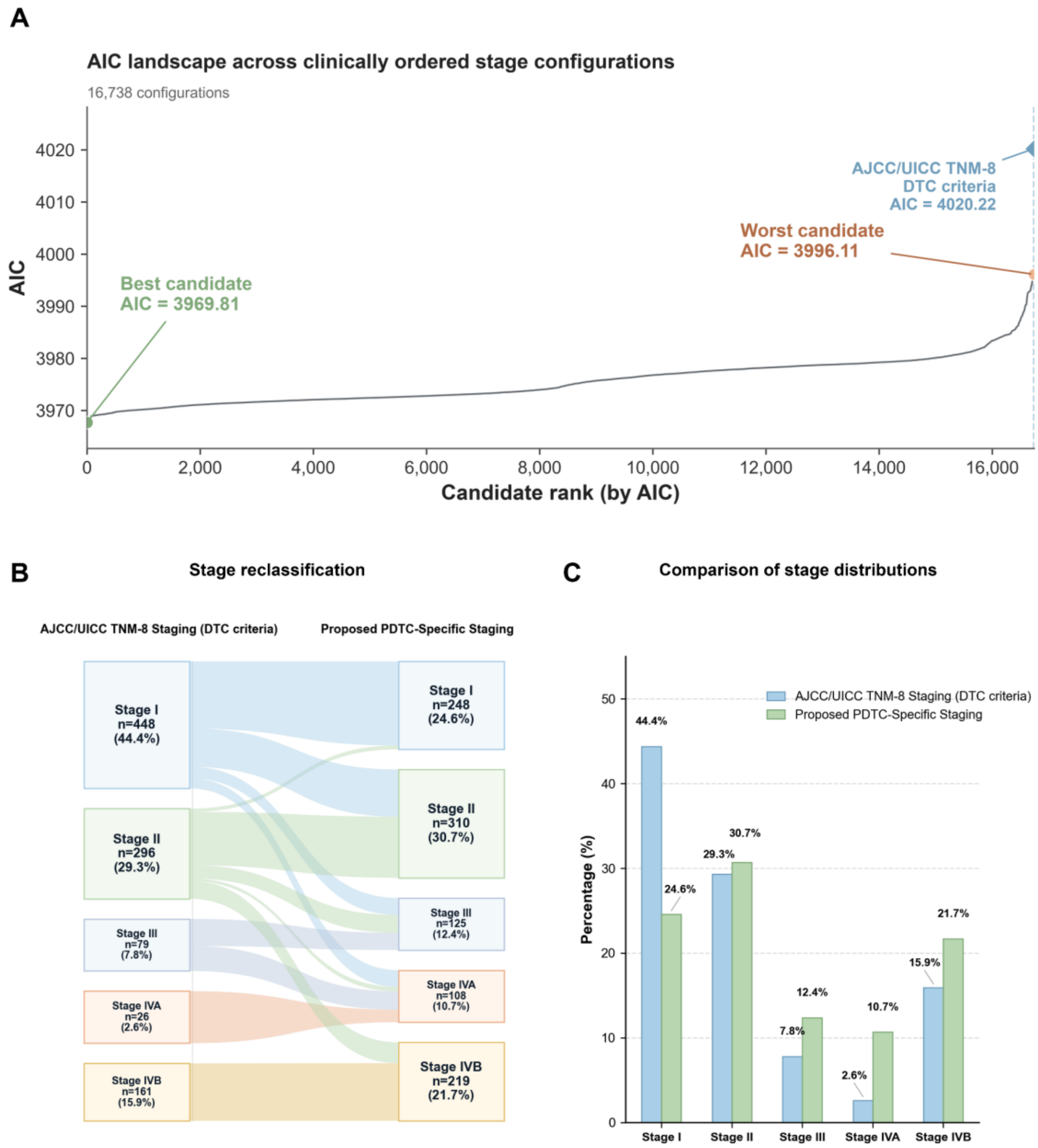

eFigure 4. Exhaustive Enumeration and Stage Redistribution for the Proposed PDTC-Specific Staging System

(A) AIC landscape of 16,738 clinically admissible T–modified N stage configurations among M0 patients with PDTC in the IATCCG cohort. Candidates are ranked by AIC from the prespecified multivariable Cox model. The minimum-AIC candidate and AJCC/UICC TNM-8 (DTC criteria) are annotated. (B) Alluvial plot showing patient-level reclassification from AJCC/UICC TNM-8 to the proposed PDTC-specific staging system in the IATCCG cohort. (C) Bar plot showing the distribution of stages under AJCC/UICC TNM-8 and the proposed staging framework in the IATCCG cohort.

© 2026 Shi X et al. *JAMA Oncology*.

A

Proposed Stage

M0 cohort; Modified N

	N0/NX	N1a	N1b	N1c
T1	I<br>aHR=1.00<br>n=83/ev=11	I<br>aHR=0.86<br>n=29/ev=3	II<br>aHR=1.43<br>n=25/ev=4	II<br>aHR=-<br>n=0/ev=0
T2	I<br>aHR=0.84<br>n=136/ev=24	II<br>aHR=1.70<br>n=24/ev=7	II<br>aHR=1.67<br>n=23/ev=5	III<br>aHR=1.74<br>n=5/ev=1
T3	II<br>aHR=1.10<br>n=202/ev=45	II<br>aHR=1.44<br>n=36/ev=10	III<br>aHR=2.07<br>n=60/ev=20	IVA<br>aHR=2.54<br>n=24/ev=9
T4a	III<br>aHR=1.95<br>n=46/ev=20	III<br>aHR=1.77<br>n=14/ev=4	IVA<br>aHR=2.67<br>n=36/ev=16	IVA<br>aHR=2.76<br>n=11/ev=5
T4b	IVA<br>aHR=2.42<br>n=15/ev=7	IVA<br>aHR=-<br>n=1/ev=0	IVA<br>aHR=3.36<br>n=19/ev=9	IVA<br>aHR=6.21<br>n=2/ev=1

B

AJCC/UICC TNM-8

M0 cohort; age-dependent DTC staging

		N0/NX	N1a	N1b
Age <55	M0	I<br>aHR=1.00<br>n=218/ev=27	I<br>aHR=2.05<br>n=57/ev=11	I<br>aHR=1.73<br>n=78/ev=15
Age ≥55	T1	I<br>aHR=0.73<br>n=27/ev=5	II<br>aHR=0.43<br>n=11/ev=1	II<br>aHR=2.79<br>n=7/ev=3
	T2	I<br>aHR=0.80<br>n=68/ev=16	II<br>aHR=1.07<br>n=16/ev=4	II<br>aHR=1.89<br>n=13/ev=3
	T3	II<br>aHR=0.91<br>n=122/ev=35	II<br>aHR=1.57<br>n=22/ev=10	II<br>aHR=1.99<br>n=47/ev=21
	T4a	III<br>aHR=1.71<br>n=33/ev=17	III<br>aHR=0.86<br>n=12/ev=2	III<br>aHR=2.81<br>n=34/ev=19
	T4b	IVA<br>aHR=2.25<br>n=14/ev=7	IVA<br>aHR=-<br>n=0/ev=0	IVA<br>aHR=2.10<br>n=12/ev=5

Stage I Stage II Stage III Stage IVA

eFigure 5. Comparison of Proposed PDTC-Specific and AJCC/UICC TNM-8 Stage Grouping Matrices

(A) Proposed PDTC-specific and (B) AJCC/UICC TNM-8 stage grouping matrices showing stage assignment, sample size, OS events, and adjusted hazard ratio (aHR) for each M0 subgroup. In the proposed staging system, M1 disease was assigned separately to Stage IVB.

© 2026 Shi X et al. *JAMA Oncology.*

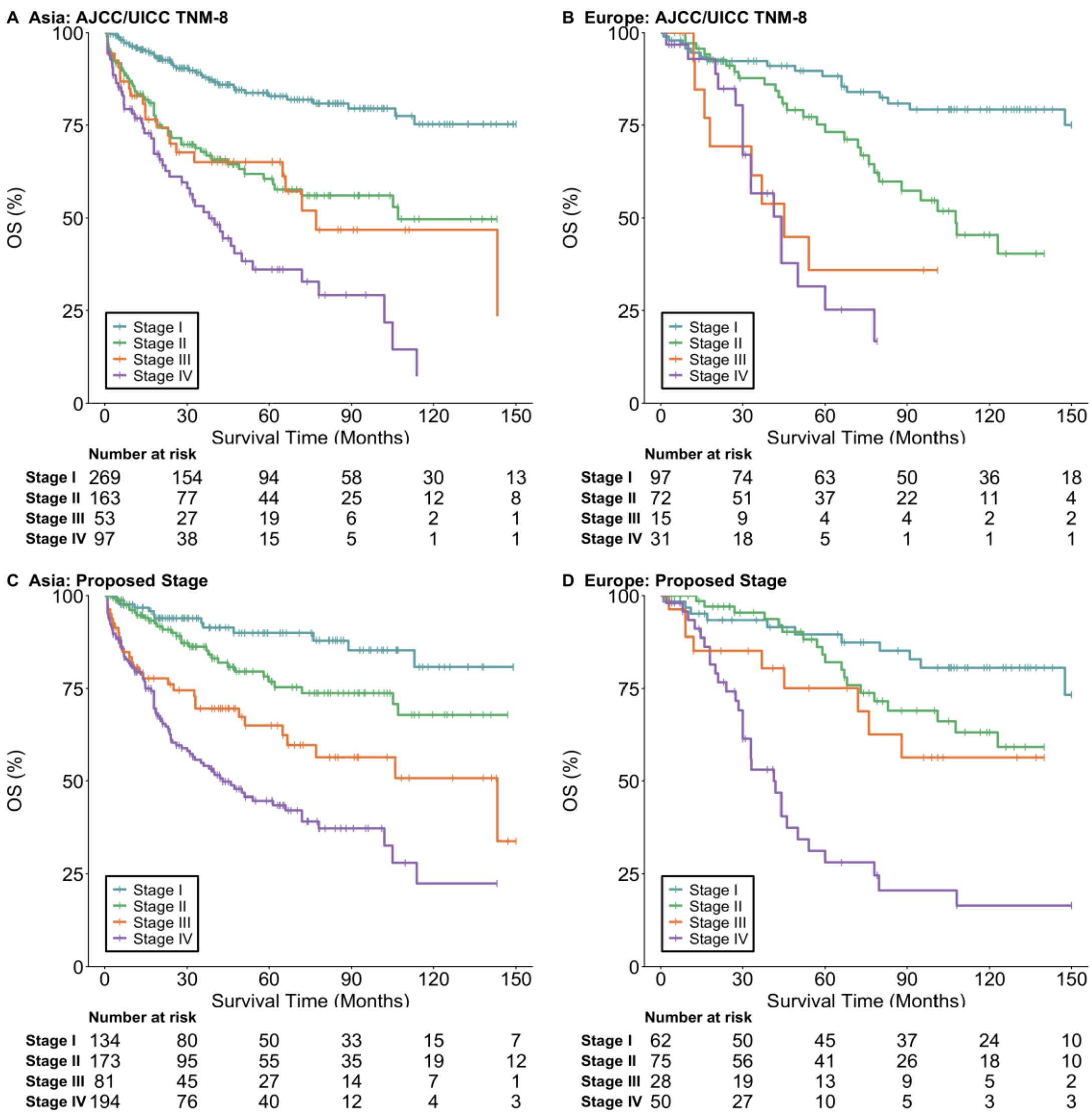

eFigure 6. Subgroup Analysis of Overall Survival Stratification in Asia and Europe

Kaplan–Meier curves show (A) Asia–AJCC/UICC TNM-8, (B) Europe–AJCC/UICC TNM-8, (C) Asia–proposed PDTC-specific staging, and (D) Europe–proposed PDTC-specific staging. Geographic regions were classified according to the United Nations geoscheme.

© 2026 Shi X et al. *JAMA Oncology*.

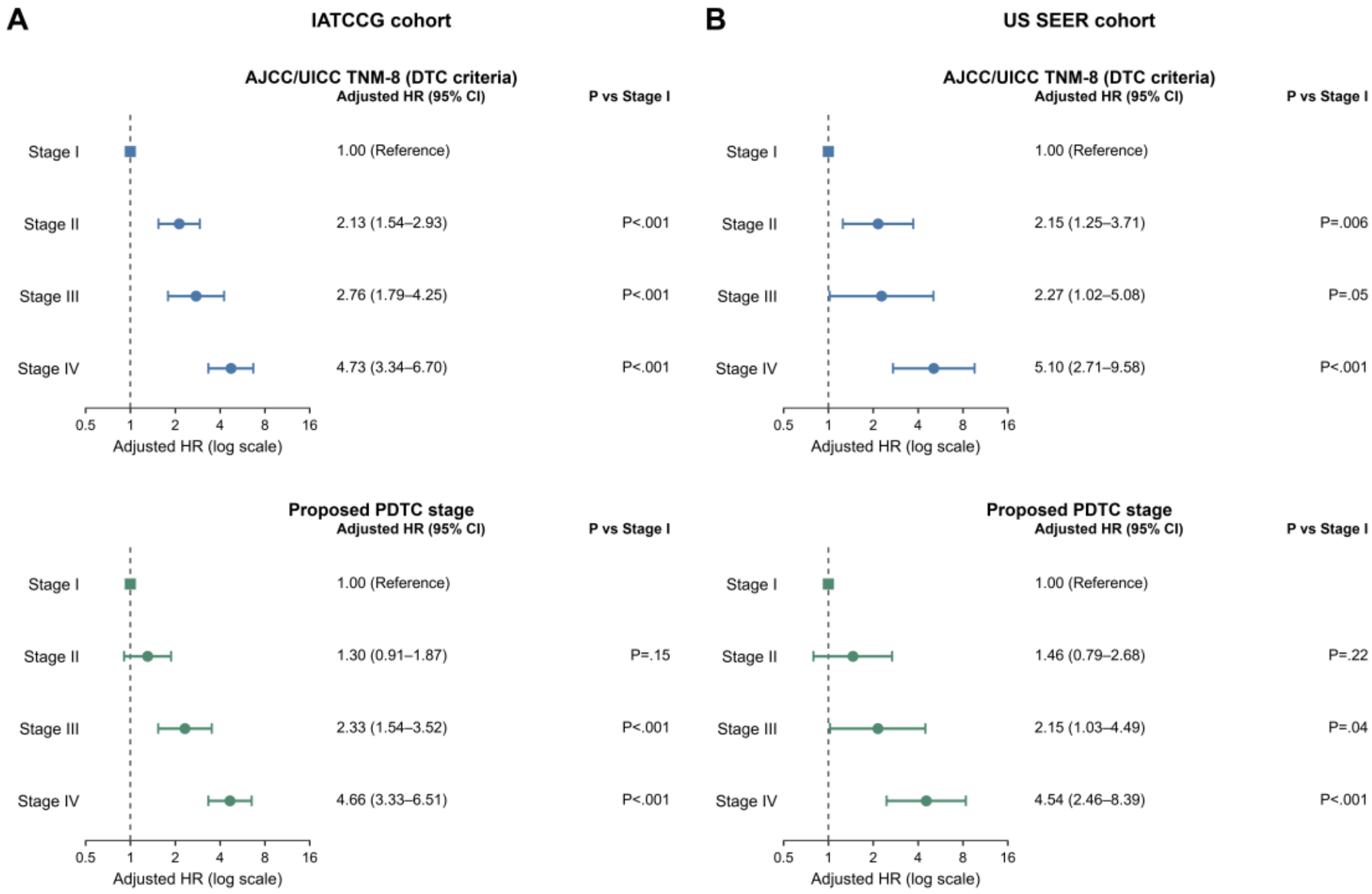

eFigure 7. Adjusted HRs and 95% CIs by Staging System

Forest plots illustrate the adjusted HRs and 95% CIs for different stages categorized by AJCC/UICC TNM-8 (DTC criteria) and the proposed PDTC staging system, using Stage I as the reference group. Analyses were adjusted for age, sex, extent of surgery, resection status, and geographic region in the IATCCG cohort, and for age, sex, and extent of surgery in the US SEER cohort.

© 2026 Shi X et al. *JAMA Oncology*.

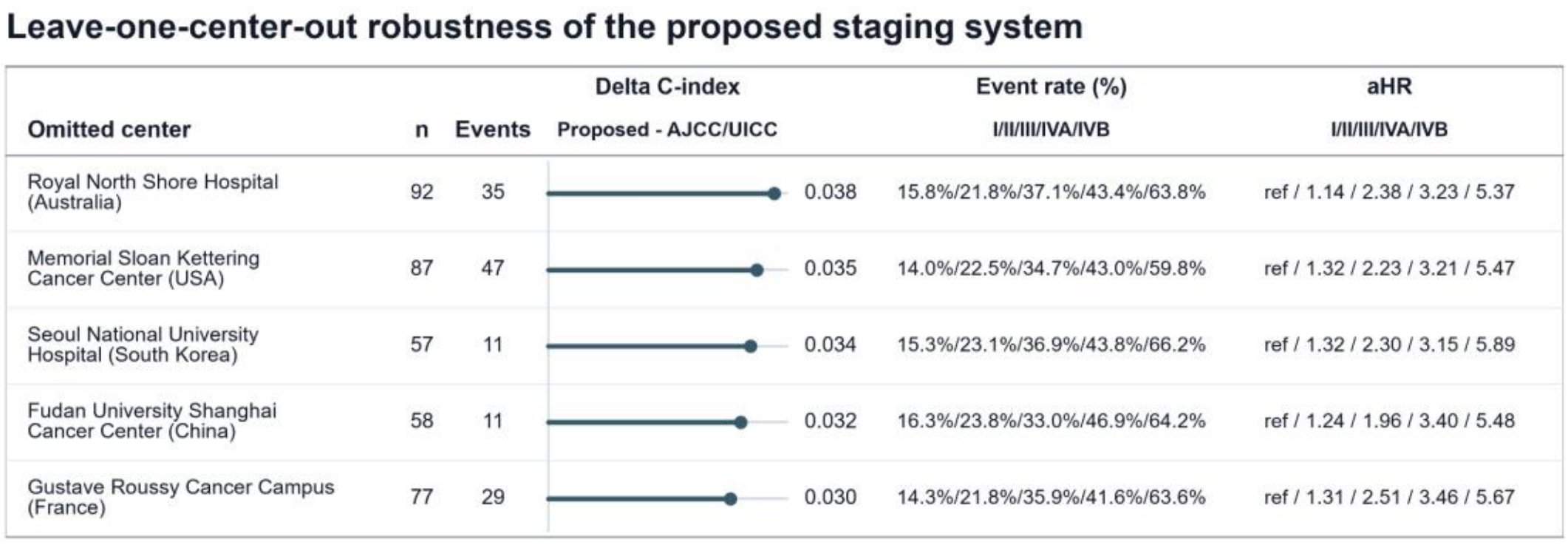

Leave-one-center-out robustness of the proposed staging system

Omitted center	n	Events	Delta C-index Proposed - AJCC/UICC	Event rate (%) I/II/III/IVA/IVB	aHR I/II/III/IVA/IVB
Royal North Shore Hospital (Australia)	92	35	0.038	15.8%/21.8%/37.1%/43.4%/63.8%	ref / 1.14 / 2.38 / 3.23 / 5.37
Memorial Sloan Kettering Cancer Center (USA)	87	47	0.035	14.0%/22.5%/34.7%/43.0%/59.8%	ref / 1.32 / 2.23 / 3.21 / 5.47
Seoul National University Hospital (South Korea)	57	11	0.034	15.3%/23.1%/36.9%/43.8%/66.2%	ref / 1.32 / 2.30 / 3.15 / 5.89
Fudan University Shanghai Cancer Center (China)	58	11	0.032	16.3%/23.8%/33.0%/46.9%/64.2%	ref / 1.24 / 1.96 / 3.40 / 5.48
Gustave Roussy Cancer Campus (France)	77	29	0.030	14.3%/21.8%/35.9%/41.6%/63.6%	ref / 1.31 / 2.51 / 3.46 / 5.67

eFigure 8. Leave-1-Center-Out Sensitivity Analysis Comparing the Proposed Staging System With AJCC/UICC TNM-8 in the IATCCG Cohort

Centers with at least 50 patients and 10 deaths were excluded one at a time, and the analyses were repeated in the remaining cohort. ΔC-index represents the difference between the proposed system and AJCC/UICC TNM-8; stage-specific event rates and aHRs are shown for the proposed system. The n and events columns indicate the number of patients and deaths in the excluded center.

© 2026 Shi X et al. *JAMA Oncology*.

A **Geographic exclusion sensitivity analysis**

Excluded geography	Excluded n	Remaining n	Deaths	ΔC-index (Proposed − AJCC8)	ΔAIC (Proposed − AJCC8)
Asia	582	428	173	0.026	-18.92
Europe	215	795	267	0.029	-39.15
China	499	511	188	0.030	-22.17

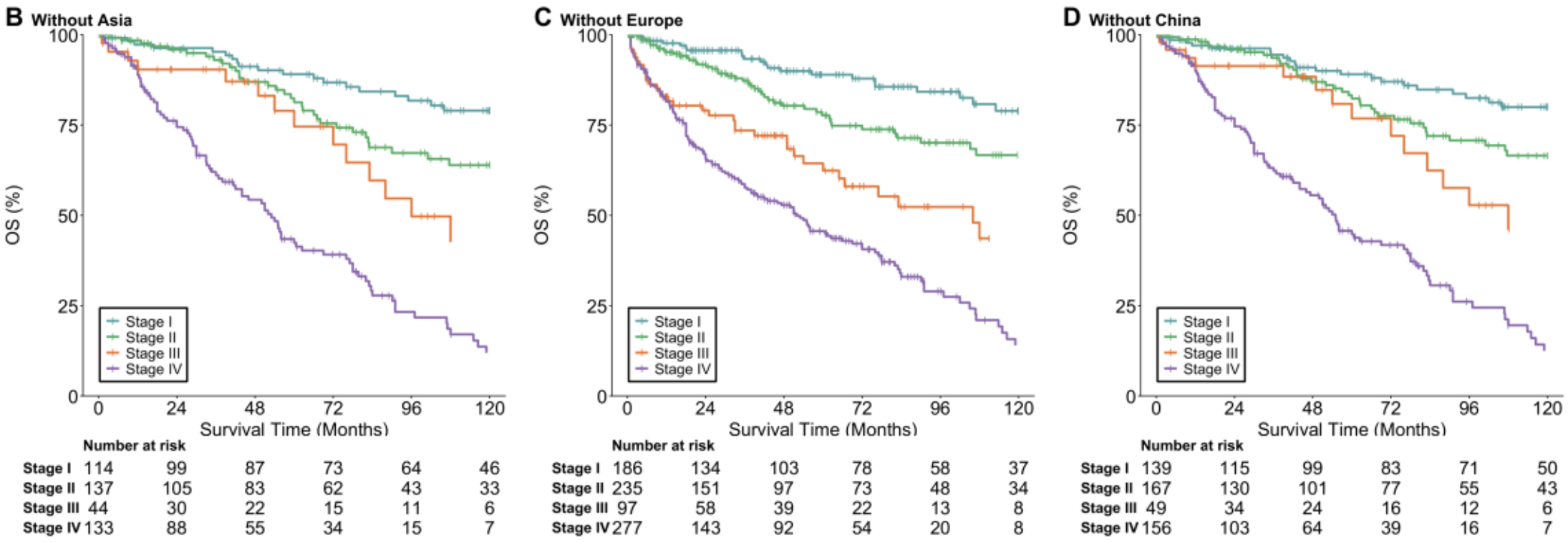

eFigure 9. Geographic Exclusion Sensitivity Analysis

(A) Differences in prognostic performance between the fixed proposed staging system and AJCC/UICC TNM-8 after excluding Asia, Europe, or China, assessed by ΔC-index and ΔAIC from adjusted Cox models. (B–D) Kaplan–Meier overall survival curves after the corresponding exclusions.

© 2026 Shi X et al. *JAMA Oncology*.

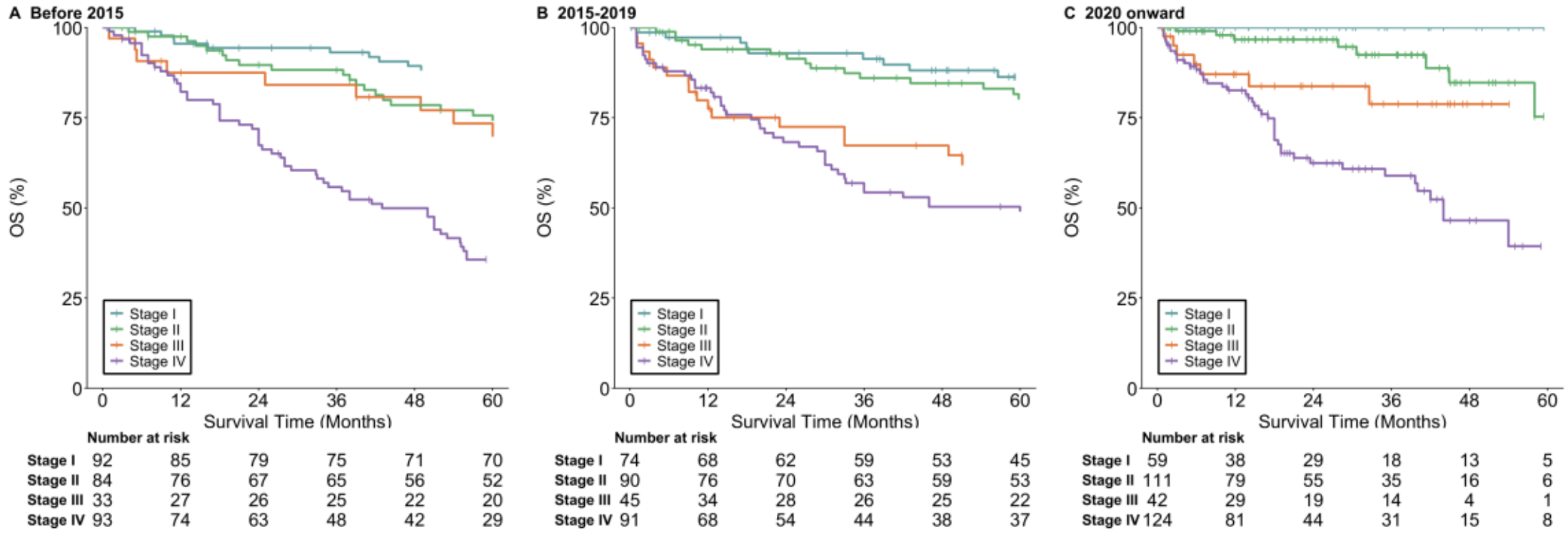

eFigure 10. Kaplan-Meier Overall Survival Curves Stratified by Calendar Year of Diagnosis

Patients were grouped as (A) before 2015, (B) during 2015–2019, and (C) from 2020 onward. The analysis included 938 patients with diagnosis dates; 72 were excluded because diagnosis dates were unavailable under institutional privacy policies.

© 2026 Shi X et al. *JAMA Oncology*.
