# Supplementary material for: Global Proposal for the TNM Staging System for Poorly Differentiated Thyroid Cancer
Source: JAMA Oncol. 2026 Nov 6:e264705. Online ahead of print. doi: 10.1001/jamaoncol.2026.4705 (PMC13744029; doi:10.1001/jamaoncol.2026.4705)
Supplement: Supplementary file 2 [file jamaoncol-e264705-s002.pdf]

Supplemental Online Content: Nonauthor Collaborators
*First name, last name, and suffix (if applicable) are required and will appear in PubMed.

*Group Name(s): International Aggressive Thyroid Cancer Collaborative Group							
***First Name and Middle Initial(s)**	***Last Name**	***Suffix (eg, Jr, III)**	Academic Degrees	Institution	Location (city, state/province, country)	Role or Contribution, eg, chair, principal investigator	Group (if more than 1 Group listed in the byline) and/or Subgroup (eg, Steering Committee)
Yulong	Wang		MD, PhD	Fudan University Shanghai Cancer Center, Department of Oncology, Shanghai Medical College, Fudan University	Shanghai, China	administrative support	
Julien	Hadoux		MD, PhD	Gustave Roussy, Université Paris Saclay; ENDOCAN-TUTHYREF Network	Villejuif, France	data collection	
Stanley B	Sidhu		MD	Royal North Shore Hospital	Sydney, Australia	data collection	
Mark S	Sywak		MD	Royal North Shore Hospital	Sydney, Australia	data collection	
Arseny A	Semenov		MD, PhD	Saint-Petersburg State University Hospital	Saint-Petersburg, Russia	data collection	
Bekir	Cakir		MD	Ankara Bilkent City Hospital, Ankara Yildirim Beyazit University Faculty of Medicine	Ankara, Turkey	data collection	
Ankui	Yang		MD	Sun Yat-Sen University Cancer Center, Sun Yat-sen University	Guangzhou, China	data collection	
Jiapeng	Huang		MD	The First Hospital of China Medical University	Shenyang, China	data collection	
Kehao	Le		MD	Sir Run Run Shaw Hospital, Zhejiang University School of Medicine	Hangzhou, China	data collection	
Weihao	Liu		MD	The First Affiliated Hospital of Zhengzhou University	Zhengzhou, China	data collection	
Zhengchao	Wei		MD	Jilin Cancer Hospital	Changchun, China	data collection	
Shitong	Yu		MD	Nanfang Hospital, Southern Medical University	Guangzhou, China	data collection	
Yanbin	Zhao		MD	Jiangsu Cancer Hospital	Nanjing, China	data collection	

Supplemental Online Content: Nonauthor Collaborators

*First name, last name, and suffix (if applicable) are required and will appear in PubMed.

***First Name and Middle Initial(s)**	***Last Name**	***Suffix (eg, Jr, III)**	Academic Degrees	Institution	Location (city, state/province, country)	Role or Contribution, eg, chair, principal investigator	Group (if more than 1 Group listed in the byline) and/or Subgroup (eg, Steering Committee)
Yajing	Zhou		MD	Union Hospital, Tongji Medical College, Huazhong University of Science and Technology	Wuhan, China	data collection	
Yu	Guo		MD	Sun Yat-Sen Memorial Hospital, Sun Yat-sen University	Guangzhou, China	data collection	
Shuping	Wu		MD	Fujian Cancer Hospital	Fuzhou, China	data collection	
Chaoqian	Liu		MD	Shanghai Changhai Hospital, Naval Medical University	Shanghai, China	data collection	
Huilei	Dong		MD	Cancer Hospital of China Medical University	Shenyang, China	data collection	
Yaying	Du		MD	Tongji Hospital, Tongji Medical College, Huazhong University of Science and Technology	Wuhan, China	data collection	
Hui	Ouyang		MD	Xiangya Hospital, Central South University	Changsha, China	data collection	
Yongbiao	Luo		MD	Affiliated Tumor Hospital of Guangxi Medical University	Nanning, China	data collection	
Cuiwei	Li		MD	The First Affiliated Hospital, Zhejiang University School of Medicine	Hangzhou, China	data collection	
Chenyi	Wang		MD	China-Japan Friendship Hospital	Beijing, China	data collection	
Kaiyi	Niu		MD	The First Affiliated Hospital with Nanjing Medical University	Nanjing, China	data collection	
Qijun	Li		MD	Peking Union Medical College Hospital	Beijing, China	data collection	
Wei	Liu		MD	Zhongshan Hospital, Fudan University	Shanghai, China	data collection	
Tongliang	Xia		MD	Qilu Hospital of Shandong University	Jinan, China	data collection	

*First name, last name, and suffix (if applicable) are required and will appear in PubMed.

***First Name and Middle Initial(s)**	***Last Name**	***Suffix (eg, Jr, III)**	Academic Degrees	Institution	Location (city, state/province, country)	Role or Contribution, eg, chair, principal investigator	Group (if more than 1 Group listed in the byline) and/or Subgroup (eg, Steering Committee)
Xindan	Zhang		MD	Shanghai Sixth People's Hospital, Shanghai Jiao Tong University School of Medicine	Shanghai, China	data collection	
Rili	Gao		MD	Guangdong Provincial People’s Hospital, Southern Medical University	Guangzhou, China	data collection	
Deguang	Kong		MD	Renmin Hospital of Wuhan University	Wuhan, China	data collection	
Jiaye	Liu		MD	West China Hospital, Sichuan University	Chengdu, China	data collection	
Anggi	Vélez-Bohórquez		MD	Grupo de Citogenética, Filogenia y Evolución de Poblaciones, Facultades de Ciencias y Facultad de Ciencias de la Salud, Universidad del Tolima	Ibagué, Colombia	data collection	
Carlos Eduardo	Jiménez-Canizales		MD	Universidad del Tolima; Universidad Nacional de Colombia	Ibagué; Bogotá, Colombia	data collection	
Maria João	Martins		MD	Hospitais Universitários de Coimbra, Unidade Local de Saúde de Coimbra	Coimbra, Portugal	data collection	
Matteo	Trevisan		MD	ASST Santi Paolo e Carlo; Department of Health Sciences, University of Milan	Milan, Italy	data collection	
Guowei	Zhang		MD	Xuzhou Central Hospital	Xuzhou, China	data collection	
Hailin	Xi		MD	Xuzhou Central Hospital	Xuzhou, China	principal investigator	
Yefeng	Cai		MD	The First Affiliated Hospital of Wenzhou Medical University	Wenzhou, China	data collection	

Supplemental Online Content: Nonauthor Collaborators
*First name, last name, and suffix (if applicable) are required and will appear in PubMed.

***First Name and Middle Initial(s)**	***Last Name**	***Suffix (eg, Jr, III)**	Academic Degrees	Institution	Location (city, state/province, country)	Role or Contribution, eg, chair, principal investigator	Group (if more than 1 Group listed in the byline) and/or Subgroup (eg, Steering Committee)
Hongzhong	Zhou		MD	The First Affiliated Hospital of Wenzhou Medical University	Wenzhou, China	principal investigator	
Anbing	Dong		MD	The Affiliated Hospital of Qingdao University	Qingdao, China	data collection	
Hui	Hua		MD	The Affiliated Hospital of Qingdao University	Qingdao, China	principal investigator	
Chao	Qin		MD	Nanjing Drum Tower Hospital, Affiliated Hospital of Medical School, Nanjing University	Nanjing, China	data collection	
Jianfeng	Sang		MD	Nanjing Drum Tower Hospital, Affiliated Hospital of Medical School, Nanjing University	Nanjing, China	principal investigator	
Gang	Li		MD	Henan Cancer Hospital, Affiliated Cancer Hospital of Zhengzhou University	Zhengzhou, China	data collection	
Shanting	Liu		MD	Henan Cancer Hospital, Affiliated Cancer Hospital of Zhengzhou University	Zhengzhou, China	principal investigator	
Hao	Pi		MD	Shanghai Tenth People’s Hospital, School of Medicine, Tongji University	Shanghai, China	data collection	
Yuan	Sheng		MD	Shanghai Tenth People’s Hospital, School of Medicine, Tongji University	Shanghai, China	principal investigator	
Xiaolong	Zhao		MD	Sichuan Provincial People’s Hospital, School of Medicine, University of Electronic Science and Technology of China	Chengdu, China	data collection	

Supplemental Online Content: Nonauthor Collaborators
*First name, last name, and suffix (if applicable) are required and will appear in PubMed.

***First Name and Middle Initial(s)**	***Last Name**	***Suffix (eg, Jr, III)**	Academic Degrees	Institution	Location (city, state/province, country)	Role or Contribution, eg, chair, principal investigator	Group (if more than 1 Group listed in the byline) and/or Subgroup (eg, Steering Committee)
Bin	Li		MD	Sichuan Provincial People's Hospital, School of Medicine, University of Electronic Science and Technology of China	Chengdu, China	principal investigator	
Jianhong	Ye		MD	The First Hospital of Jiaxing, Jiaxing University	Jiaxing, China	data collection	
Jian	Tang		MD	The First Hospital of Jiaxing, Jiaxing University	Jiaxing, China	principal investigator	
Huaguo	Wu		MD	Shandong Cancer Hospital and Institute, Shandong First Medical University and Shandong Academy of Medical Sciences	Jinan, China	data collection	
Wanjun	Chen		MD	Shandong Cancer Hospital and Institute, Shandong First Medical University and Shandong Academy of Medical Sciences	Jinan, China	principal investigator	
Qingxia	Wang		MD	Hunan Cancer Hospital & The Affiliated Cancer Hospital of Xiangya School of Medicine, Central South University	Changsha, China	data collection	
Xiaowei	Peng		MD	Hunan Cancer Hospital & The Affiliated Cancer Hospital of Xiangya School of Medicine, Central South University	Changsha, China	principal investigator	
Jiahao	Pan		MD	Shanghai Changzheng Hospital, Naval Medical University	Shanghai, China	data collection	
Wei	Zhang		MD	Shanghai Changzheng Hospital, Naval Medical University	Shanghai, China	principal investigator	

Supplemental Online Content: Nonauthor Collaborators
*First name, last name, and suffix (if applicable) are required and will appear in PubMed.

***First Name and Middle Initial(s)**	***Last Name**	***Suffix (eg, Jr, III)**	Academic Degrees	Institution	Location (city, state/province, country)	Role or Contribution, eg, chair, principal investigator	Group (if more than 1 Group listed in the byline) and/or Subgroup (eg, Steering Committee)
Wenxin	Zhao		MD	Fujian Medical University Union Hospital	Fuzhou, China	principal investigator	
Mengxin	Zhang		MD	The Second Affiliated Hospital of Dalian Medical University	Dalian, China	data collection	
Na	Li		MD	The Second Affiliated Hospital of Dalian Medical University	Dalian, China	principal investigator	
Tingting	Zhang		MD	The First Affiliated Hospital of University of Science and Technology of China	Hefei, China	data collection	
Xiaopeng	Ma		MD	The First Affiliated Hospital of University of Science and Technology of China	Hefei, China	principal investigator	
Yuntao	Song		MD	Peking University Cancer Hospital and Institute	Beijing, China	data collection	
Bin	Zhang		MD	Peking University Cancer Hospital and Institute	Beijing, China	principal investigator	
Fu	Luo		MD	Daping Hospital, Army Medical University	Chongqing, China	data collection	
Yan	Xu		MD	Daping Hospital, Army Medical University	Chongqing, China	principal investigator	
Chen	Chen		MD	Huashan Hospital, Fudan University	Shanghai, China	data collection	
Yiting	Jin		MD	Huashan Hospital, Fudan University	Shanghai, China	principal investigator	
Lu	Zhang		MD	Affiliated Hospital of Inner Mongolia Medical University	Hohhot, China	data collection	
Junwen	Bai		MD	Affiliated Hospital of Inner Mongolia Medical University	Hohhot, China	principal investigator	
Yang	Li		MD	Shanghai General Hospital Affiliated to Shanghai Jiao Tong University School of Medicine	Shanghai, China	data collection	

*First name, last name, and suffix (if applicable) are required and will appear in PubMed.

***First Name and Middle Initial(s)**	***Last Name**	***Suffix (eg, Jr, III)**	Academic Degrees	Institution	Location (city, state/province, country)	Role or Contribution, eg, chair, principal investigator	Group (if more than 1 Group listed in the byline) and/or Subgroup (eg, Steering Committee)
Xing	Sun		MD	Shanghai General Hospital Affiliated to Shanghai Jiao Tong University School of Medicine	Shanghai, China	principal investigator	
Chen	Li		MD	Peking University People's Hospital	Beijing, China	data collection	
Kewei	Jiang		MD	Peking University People's Hospital	Beijing, China	principal investigator	
Xuhang	Zhu		MD	Zhejiang Cancer Hospital, Hangzhou Institute of Medicine, Chinese Academy of Sciences	Hangzhou, China	data collection	
Jinbiao	Shang		MD	Zhejiang Cancer Hospital, Hangzhou Institute of Medicine, Chinese Academy of Sciences	Hangzhou, China	principal investigator	
Lunhua	Guo		MD	Harbin Medical University Cancer Hospital	Harbin, China	data collection	
Susheng	Miao		MD	Harbin Medical University Cancer Hospital	Harbin, China	principal investigator	
Nan	Feng		MD	Anhui Provincial Cancer Hospital, West District of The First Affiliated Hospital of University of Science and Technology of China	Hefei, China	data collection	
Xucai	Zheng		MD	Anhui Provincial Cancer Hospital, West District of The First Affiliated Hospital of University of Science and Technology of China	Hefei, China	principal investigator	
Erica	Solaroli		MD	Endocrinology Unit - Maggiore Hospital	Bologna, Italy	data collection	

Supplemental Online Content: Nonauthor Collaborators
*First name, last name, and suffix (if applicable) are required and will appear in PubMed.

***First Name and Middle Initial(s)**	***Last Name**	***Suffix (eg, Jr, III)**	Academic Degrees	Institution	Location (city, state/province, country)	Role or Contribution, eg, chair, principal investigator	Group (if more than 1 Group listed in the byline) and/or Subgroup (eg, Steering Committee)
Xiaohua	Tai		MD	The Second Hospital of Jiaxing, Jiaxing University	Jiaxing, China	data collection	
Xueyong	Zheng		MD	The Second Hospital of Jiaxing, Jiaxing University	Jiaxing, China	principal investigator	
Jiawei	Feng		MD	The First People’s Hospital of Changzhou	Changzhou, China	data collection	
Yong	Jiang		MD	The First People’s Hospital of Changzhou	Changzhou, China	principal investigator	
Yuyuan	Ma		MD	Suzhou Municipal Hospital	Suzhou, China	data collection	
Xiaomin	Gu		MD	Suzhou Municipal Hospital	Suzhou, China	principal investigator	
Chunlian	Li		MD	Northern Jiangsu People's Hospital Affiliated to Yangzhou University	Yangzhou, China	data collection	
Deyuan	Fu		MD	Northern Jiangsu People's Hospital Affiliated to Yangzhou University	Yangzhou, China	principal investigator	
Liang	Sun		MD	The First Affiliated Hospital of Soochow University	Suzhou, China	data collection	
Enqiao	Yu		MD	The First Affiliated Hospital of Soochow University	Suzhou, China	principal investigator	
Qin	Li		MD	Yijishan Hospital, The First Affiliated Hospital of Wannan Medical College	Wuhu, China	data collection	
Mengshi	Chi		MD	The Seventh Affiliated Hospital, Sun Yat-Sen University	Shenzhen, China	data collection	
Kepeng	Zhu		MD	The Affiliated Nanchong Central Hospital of North Sichuan Medical College	Nanchong, China	data collection	

Supplemental Online Content: Nonauthor Collaborators

*First name, last name, and suffix (if applicable) are required and will appear in PubMed.

***First Name and Middle Initial(s)**	***Last Name**	***Suffix (eg, Jr, III)**	Academic Degrees	Institution	Location (city, state/province, country)	Role or Contribution, eg, chair, principal investigator	Group (if more than 1 Group listed in the byline) and/or Subgroup (eg, Steering Committee)
Junyuan	Lv		MD	Affiliated Hospital of Zunyi Medical University	Zunyi, China	data collection	
Shibo	Liang		MD	Daqing Longnan Hospital	Daqing, China	data collection	
Yuantong	Shi		MD	The Fifth People's Hospital of Qinghai Province	Xining, China	principal investigator	
Qiulan	Chen		MD	Jiangsu Taizhou Traditional Chinese Medicine Hospital	Taizhou, China	principal investigator	
Chunlan	He		MD	Yangzhou Hospital of Traditional Chinese Medicine	Yangzhou, China	data collection	
Linkun	Zhong		MD	Zhongshan City People's Hospital	Zhongshan, China	principal investigator	
Ming	Gao		MD	Tianjin Union Medical Center, The First Affiliated Hospital of Nankai University	Tianjin, China	administrative support	
Ruochuan	Cheng		MD	First Affiliated Hospital of Kunming Medical University	Kunming, China	administrative support	
Minghua	Ge		MD	Zhejiang Provincial People's Hospital, Hangzhou Medical College	Hangzhou, China	administrative support	
Shaoyan	Liu		MD	National Cancer Center/National Clinical Research Center for Cancer/Cancer Hospital, Chinese Academy of Medical Sciences and Peking Union Medical College	Beijing, China	administrative support	

Supplemental Online Content: Nonauthor Collaborators
*First name, last name, and suffix (if applicable) are required and will appear in PubMed.

***First Name and Middle Initial(s)**	***Last Name**	***Suffix (eg, Jr, III)**	Academic Degrees	Institution	Location (city, state/province, country)	Role or Contribution, eg, chair, principal investigator	Group (if more than 1 Group listed in the byline) and/or Subgroup (eg, Steering Committee)
Changming	An		MD	National Cancer Center/National Clinical Research Center for Cancer/Cancer Hospital, Chinese Academy of Medical Sciences and Peking Union Medical College	Beijing, China	administrative support	
