# Supplementary material for: Global Proposal for the TNM Staging System for Poorly Differentiated Thyroid Cancer
Source: JAMA Oncol. 2026 Nov 6:e264705. Online ahead of print. doi: 10.1001/jamaoncol.2026.4705 (PMC13744029; doi:10.1001/jamaoncol.2026.4705)
Supplement: Supplementary file 3 [file jamaoncol-e264705-s003.pdf]

Data Sharing Statement

Shi. Global Proposal for the TNM Staging System for Poorly Differentiated Thyroid Cancer. *JAMA Oncol*. Published November 06, 2026. doi:10.1001/jamaoncol.2026.4705

Data
Data available: No

Additional Information
Explanation for why data not available: Deidentified data supporting the findings of this Article may be available from the corresponding author (Yu Wang, neck130@sina.com) after publication upon reasonable written request and required institutional or ethics approval. Data will be shared only for scientifically appropriate purposes under conditions protecting participant confidentiality. Consent forms, ethics submissions, data transfer agreements, and other administrative or source documents will not be shared because they may compromise patient privacy or institutional confidentiality.
